# Perspectives on Gender in Science, Technology, and Innovation: A Review of Sub-Saharan Africa's Science Granting Councils and Achieving the Sustainable Development Goals

**DOI:** 10.3389/frma.2022.814600

**Published:** 2022-04-11

**Authors:** Jose C. Jackson, Jane G. Payumo, Amy J. Jamison, Michael L. Conteh, Petronella Chirawu

**Affiliations:** ^1^Alliance for African Partnership, Michigan State University, East Lansing, MI, United States; ^2^Southern Africa Research and Innovation Management Association (SARIMA), Pretoria, South Africa; ^3^MSU AgBioResearch, Michigan State University, East Lansing, MI, United States

**Keywords:** gender, Africa, Science Granting Council Initiative (SGCI), sustainable development goals, science technology and innovation

## Abstract

Africa's focus on science, technology, and innovation (STI) has grown over the last decade, with emerging examples of good practice. There are however numerous challenges to sustainable development in Africa; for example, inequalities within and among African countries are rising and enormous disparities of opportunity, wealth, and power persist. While policy makers and organizations have put increasing emphasis on integrating gender into STI policies and initiatives as a means to achieve gender equality for all women and girls, inequality remains a key challenge to continental sustainable development. STI funders such as the Science Granting Councils (SGCs) in Africa are key players in national innovation systems. They advise and facilitate policy and program development, disburse funds, build research capacity, set and monitor research agendas, manage bilateral and multilateral STI agreements, and assess the communication, uptake, and impact of research. They, therefore, have a major role to play in enabling countries to achieve SDG5. This study assessed the current actions in gender mainstreaming across the SGCs and the status of gender research and collaboration in participating countries. Our findings provide evidence of uneven progress in promoting gender equality in the operations of the SGCs, including funding research and promoting the integration of gender dimensions in research content and curricula. All SGCs emphasized national commitments to gender, and the importance of gender in STI, but acknowledged that at the structural and institutional levels there was a misalignment between policy and practice. As expected, more men than women were employed across most levels at the SGCs and held positions of seniority and decision making. Most of the SGCs had very limited or no gender-related funding programs to promote gender and STI or to eliminate the barriers that women scholars face. This resulted in persistent inequalities in who received funding, the size of the grants they received, and in the knowledge production, collaboration, and the impact on their country's gender-related research. These findings suggest that SGCs need to strengthen their actions to mainstream gender if they are to achieve success with SDG5.

## Introduction

It is well-documented that science, technology, and innovation (STI) drive countries' economic growth (Organization for Economic Cooperation and Development (OECD), 2005; Kraemer-Mbula and Wamae, 2010; New Partnership for Africa's Development (NEPAD), 2014) and contribute to improving the well-being of individuals and their communities (Schiebiger, 2010). STI investment is an important means for countries to improve productivity and competitiveness and to create decent work opportunities (UNESCO, 2010). STI-driven economies emphasize research and development (R&D), entrepreneurship, and innovation (Raghupathi and Raghupathi, 2019). The African Union's Science, Technology, and Innovation Strategy for Africa (STISA-2024) and its socio-economic transformation plan for the continent—*Agenda 2063* recognized at the highest level the critical role STI plays in Africa's economic development (African Union Commission (AU), 2014, 2015). Continuous investment in STI was presented as the only way to bridge the gap between Africa and more developed countries so that Africa could create wealth, peace, and sustainable development for its people.

STISA-2024 and Agenda 2063 made urgent policy recommendations that included:

1. Member states should develop, implement and monitor STI policies and strategies at national, regional, and continental levels, and identify best practices across contexts;
2. National ministries of science and technology in African countries should be established to take the lead in STI policy assessment and evaluation in their countries;
3. STI funding should be increased to at least 2% of GDP;
4. Public collaboration with the private sector should be stimulated and encouraged and international partnerships deepened;
5. African human capital should be maximized and strategies to attract those in the diaspora to contribute to Africa through mentorship, and capacity development programs to inspire the next generation of African scientists should be put in place;
6. Education policies, structures, and curriculum should be reviewed to better address the continent's STEM education and entrepreneurship needs; and
7. Continental policies to address STI gender disparity should be developed to mainstream gender into STI policies and strategies; promote access of girls and women to STEM education at all levels of the education system; establish collaborative networks and mentorship programs for women scientists; improve conditions for the recruitment, retention, and advancement of women in STI fields; and launch national and regional campaigns to raise awareness about the contributions of women to STI to overcome existing gender stereotypes among scientists, policymakers, and society in general.

Africa's STI policy focus has grown since STISA-2024 and Agenda 2063, with emerging examples of good practice. At a regional level, the Southern African Development Community (SADC) established a department of STI within its Directorate of Industrial Development and Trade (IDT). That department has worked for many years to put national STI policies in place (Kraemer-Mbula and Scerri, 2016). The East African Science and Technology Commission (EASTECO) was established to promote and coordinate the development, management, and application of STI in the East African Community (EAC) and focused its efforts on harmonizing national STI policies that already exist (Urama et al., 2016). While the STI community in West Africa appeared to make faster progress in countries like Nigeria, Ghana, and Senegal than elsewhere, there were still some countries of low progress, especially those that had relatively higher incidences of political instability, and poor national governance (UNESCO, 2013). According to the UNESCO Science Report (UNESCO, 2021), some countries in Africa including South Africa and Mauritius had attained gender parity, at least in terms of numbers; while others like Senegal significantly increased their share of women researchers. However, there were serious data gaps as sex-disaggregated data on researchers was not collected regularly by most countries in Africa. Promising developments had been observed overall in important S&T indicators in Africa, including scientific production, investment in R&D in terms of funding and infrastructure, quality of research, and creating centers of research excellence (Soete et al., 2015; Tijssen and Kraemer-Mbula, 2017). There were however, still challenges, notable of which is that women were a minority in digital information technology, computing, physics, mathematics and engineering, the fields that are driving the Fourth Industrial Revolution and, thus, many of the jobs of tomorrow (UNESCO, 2021).

One notable development supporting STI was the establishment of Science Granting Councils (SGCs), also known as funding agencies or research councils, across the continent. Essentially, they are national-level public or semi-public organizations that act as intermediaries between the state and the research community, where they define and execute a significant part of the state's science policy (Mouton et al., 2015; Chataway et al., 2019). SGCs perform several crucial functions that contribute to the effective and efficient functioning of STI systems and are essential actors in national systems of innovation. These include disbursing funds for R&D; building research capacity through appropriate scholarships and bursaries; setting and monitoring research agendas and priorities; advising on science, technology, and innovation policies; managing bilateral and multilateral S&T agreements; assessing the communication, uptake, and impact of publicly funded research and many more (Mouton et al., 2015).

A multi-country 5-year initiative called the Science Granting Council Initiative (SGCI) in sub-Saharan Africa contributed to strengthening the capacities of the SGCs in order to support research and evidence-based policies that will contribute to economic and social development in Sub-Saharan Africa (Mouton et al., 2015; International Development Research Center (IDRC), 2018). The objectives of the SGCI are to strengthen the ability of participating councils to manage research; design and monitor research programs based on the use of robust STI indicators; support knowledge exchange with the private sector; and establish partnerships between science granting councils and other science system actors. There were 15 SGCs with similar mandates participating in phase 1 of the SGCI (Table 1). The SGCI is jointly funded by the United Kingdom's Foreign, Commonwealth & Development Office (FCDO), Canada's International Development Research Centre (IDRC), and South Africa's National Research Foundation (NRF).

**Table 1:** Institutions participating in the Science Granting Councils Initiative (SGCI).

**Southern Africa**	**East Africa**	**West Africa**
Namibia (NAM) - National Commission on Research Science and Technology	Uganda (UGA)- Uganda National Council for Science and Technology	Senegal (SEN)- Ministère de l'Enseignement supérieur, de la Recherche et de I'Innovation
Malawi (MWI)- National Commission for Science and Technology	Kenya (KEN)- National Research Fund National Commission of Science and Technology and Innovation	Burkina Faso (BFA)- Programme d'Appui Stratégique à la Recherche Scientifique
Mozambique (MOZ)- Fundo Nacional de Investigação	Tanzania (TZA)- Tanzania Commission for Science and Technology	Côte d'Ivoire (CIV)- Programme d'Appui Stratégique à la Recherche Scientifique
Zimbabwe (ZWE)- Research Council of Zimbabwe	Ethiopia (ETH)- Ministry of Science and Technology	Ghana (GHA)- Ministry of Environment, Science, Technology, and Innovation
Zambia (ZMB)- National Science and Technology Council	Rwanda (RWA)- National Council for Science and Technology	
Botswana (BWA)- Ministry of Tertiary Education, Research, Science and Technology		

A recent analysis of the SGCs looked at the trends in investment in science as well as their role in the production, distribution, and consumption of knowledge, and how these shape and are shaped by different political economies (Chataway et al., 2017, 2019). The study concluded that building and strengthening transformative STI systems in Africa is as much a political and economic challenge as it is technical. Current political economy contexts, whether national, regional or global, condition the ways STI systems in Africa evolve, the goals they prioritize, and which STI system actors secure economic benefits and power (Chataway et al., 2017, 2019). One of the cross-cutting issues in the political economy analysis that does not seem to be receiving active attention within SGCs is gender. According to Chataway et al. (2017), gender appears to be either not considered important, recognized or properly articulated, or, further still, considered as a priority by the SGCs. This is despite the fact that across most African countries, there is a persistent underrepresentation of women in STI from primary through tertiary education to employment in different sectors; and there are persisting gender biases in society and research institutions relating to STI knowledge production which limit STI's potential benefits to societies (Chataway et al., 2017). Further to this, donors, policymakers, researchers, and others have increasingly voiced the need for incorporating gender perspectives that take into account gender-based differences in access, status, and power when examining an issue, action, research question, or policy, into research and evidence-based policy development (Parpart et al., 2000; World Health Organisation, 2002; Harding, 2009; Zosuls et al., 2011; Denney, 2015; European Commission (EC), 2016; Escobar et al., 2017; United Nations Women, 2018a,b.

Many recent reports confirm a global call for integrating gender into the operations of STI funders. The Global Research Council (GRC) in its scoping study on equality and status of women in research, outlined the experiences, models, and gender equality in practice at the country or institutional levels for SGCs around the world including those in Africa (Global Research Council (GRC), 2016a,b). The reports from this study assert that increasing diversity on research teams and gender integration in research are essential tools for increasing the quality of research and its potential for addressing global challenges. This GRC study was followed by a global commitment to gender equality at the GRC's 2017 Annual Meeting, which was detailed in their “Statement of Principles and Actions Promoting the Equality and Status of Women in Research” (Global Research Council (GRC), 2017).

A number of studies on gender parity in higher education institutions (HEIs) globally suggest that challenges around women obtaining doctorates in science, leading research and advancing in their careers still persists (Mauleón et al., 2008; Airini et al., 2011; L'Oréal Foundation, 2018; Fitzgerald, 2020; Fox, 2020). This is true in many African HEIs (Maphalala and Mpofu, 2017; Prozesky and Mouton, 2019; Adefuye et al., 2020) and has led to some efforts to transform gender cultures within HEIs in Africa [Association for the Development of Education in Africa, 2006; Association for the Development of Education in Africa (ADEA), 2015]; however the process to real change remains slow. The perspective of women researchers on the barriers and stereotypes they face, in particular their pre-determined roles in the family, home, and the research environment have been highlighted. Most of the women researchers reported feeling more pressure to handle home and social obligations, while aspiring men research leaders did not seem to have these constraints. They acknowledged needing more support to build confidence in their abilities to be a research leader and there was a need to establish programs so that they are not unduly disadvantaged. They recommended that culture change had to begin at the early stages of the education pipeline to enable girls and young women to feel able to take up challenges [Association for the Development of Education in Africa (ADEA), 2015; Daniels et al., 2015; Maphalala and Mpofu, 2017; Moodly and Toni, 2017; Beaudry et al., 2018; Prozesky and Beaudry, 2019; Liani et al., 2020; Jackson et al., 2022].

The objective of this study was to explore the role and activities of SGCs as potential change agents or catalysts in supporting gender and STI and document how their actions have been affected by political economic factors in their countries. It contributes to the ongoing and increasing debate about STI's impact on gender equality, which is central to the achievement of the 2030 Agenda and Sustainable Development Goals (SDG). Specifically, it focuses on evaluating the gender mainstreaming efforts and initiatives across the SGCs, the status of gender research, and research collaboration in participating countries. It also examines the role that the political economic context in select African countries has and continues to have on gender and inclusive practices in STI as well as the role gender-inclusive STI systems can play in transforming these contexts.

The paper is presented in five sections. The first section is an introduction to the study and lays out sub-Saharan Africa's progress in STI, an overview of the SGCI, and brief findings of a previous study on the political economy analysis of the SGCs including the dearth of policy and research activity and programming on gender and STI. In the second section we introduce the approach, conceptual framework, and methodology used for data collection and analysis while the third and fourth sections include the presentation of the desktop reviews, focus group discussion, and bibliometric analysis results. In the final section, we present implications of our findings and conclude by charting proposed improvements for strengthening gender and STI policy and funding programs at participating councils in the SGCI, including additional research questions that can be explored for future study.

## Conceptual Framework

**Figure 1:**
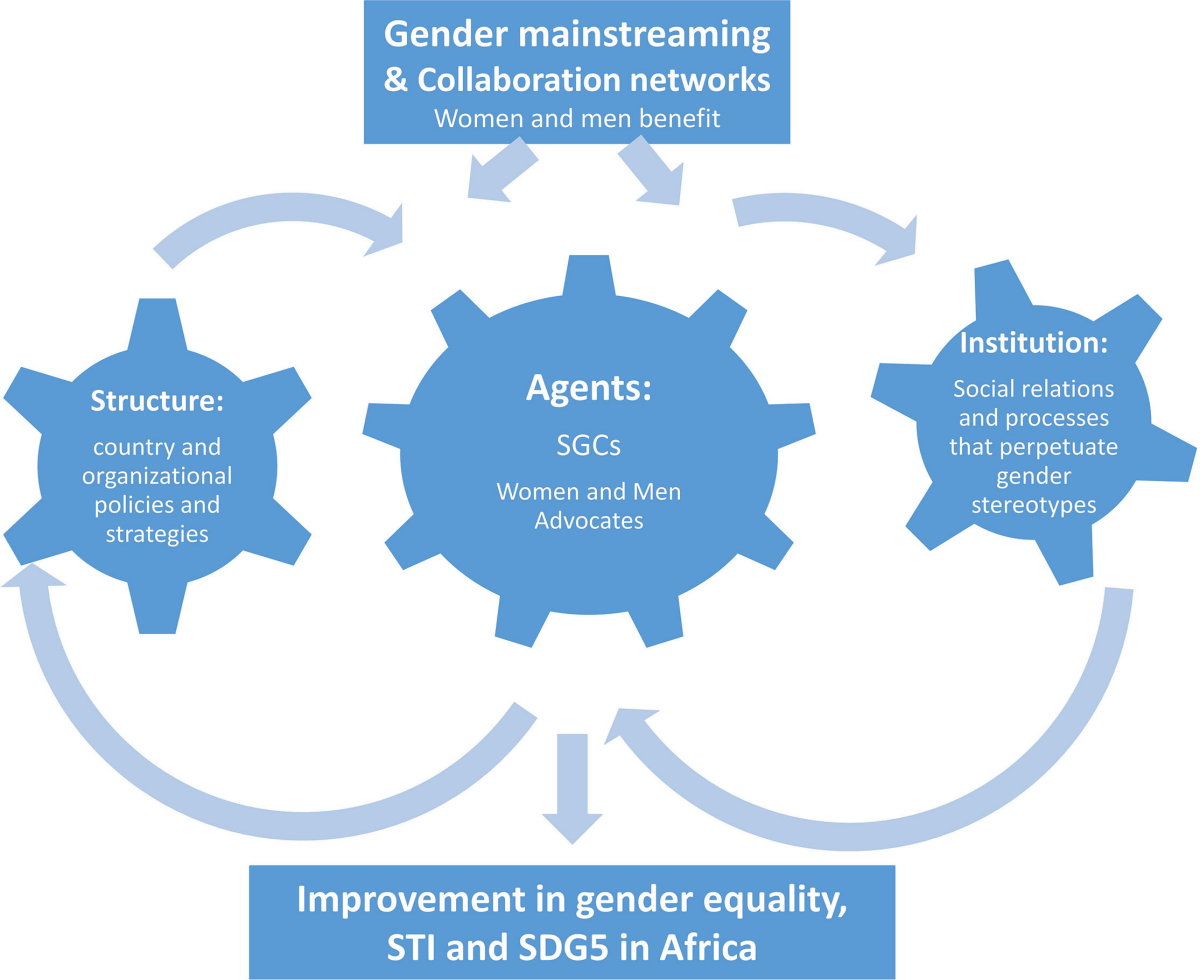
Political economy drivers of change, gender mainstreaming, and collaboration conceptual approaches for understanding how the sub-Saharan Science Granting Council Initiative (SGCI) could improve STI, gender equality, and SDG5 implementation in Africa. Adapted from Department for International Development Department for International Development (DFID) (2019), the European Institute for Gender Equality (EIGE) (2016), and Lee and Schottenfeld (2014).

This study is informed by multiple concepts including the Department for International Development (DFID) political economy (PE) Drivers of Change (DoC) as well as gender mainstreaming approaches and collaboration to address the factors that can create incentives for change around gender, STI, and making progress on SDG5 in Africa. Political economy analysis is concerned with the interaction of political and economic processes in a society: the distribution of power and wealth between different groups and individuals, and the processes that create, sustain and transform these relationships over time. The DFID PE DoC concept examines how structures, institutions, and agents can effect change in these processes and relationships [Department for International Development (DFID), 2019]. According to the United Nations (UN) (1997), gender mainstreaming is the process of assessing the implications for women and men of any planned action, including legislation, policies, or programs, in all political, economic, and societal spheres so that women and men benefit equally, and inequality is not perpetuated. This conceptual approach as illustrated in Figure 1, considers the dynamic interaction between gender mainstreaming within the context of the three components in the DFID PE DoC, and collaboration to understand how they might influence change in gender, STI, and SDG5 using the SGCI as a case study.

The first component in the DFID PE DoC concept is *structures*, defined as the systems level, long-term contextual factors including country-level or organizational policies and strategies that are generally deeply embedded and not readily influenced. The second component is *institutions*, which can be formal in the sense of rules and laws, or informal in the sense of political, social, and cultural norms and are typically the institutional social relations processes that perpetuate gender inequities and stereotypes in many African countries. The institutional factors are more susceptible to change in the medium term than structural features. The third component is the *agents*, which refers to individuals and organizations pursuing interests, such as political leaders, civil servants, political parties, private sector, trade unions, academia, civil society groups, foreign governments, regional organizations, donors, and multinational corporations.

In the conceptual framework (Figure 1), we highlighted the interaction between gender mainstreaming and the multiple levels (structures, institutions, and agents) of the DFID PE DoC. Gender mainstreaming is a process that promotes gender equality, encapsulates many of the well-studied tensions and dilemmas in feminist theory and practice and provides a new focus for debates on how to move both theories and practice forward (Beveridge et al., 2000; Behning et al., 2001; Mazey, 2001; Verloo, 2001; Woodward, 2003; Walby, 2005). A key aspect of gender mainstreaming is that it does not look at women in isolation but looks at women and men (and the relationships between them)—both as agents in the development process and as its beneficiaries. Significantly, gender mainstreaming takes as its starting point a thorough and rigorous analysis of the development situation (structural features) and seeks to address the institutional environment in which the policies and programs are developed and implemented, gender-differentiated needs and priorities, as well as gender inequalities in terms of opportunities and outcomes (Council of Europe, 1998; Neimanis, 2001; Walby, 2005, 2011; Schiebinger and Schraudner, 2011; United Nations, 2011). Since gender issues differ by country, region, and concrete situation while at the same time are impacted by national and international processes, context-relevant strategies and programming are important to ensure that the structures are sensitive to particularities of men and women's lives and seek to reduce gender-based exclusion and stereotypes to guarantee equal treatment of both men and women (Council of Europe, 1998; Neimanis, 2001). Sufficient technical capacity and human resources (agents) are core components for successfully mainstreaming gender into organizations' practices and policies. Organizations must be able to understand gender-related concepts, be able to set goals, targets, and key indicators for achieving gender equality, and have the capacity to collect and monitor data and evaluate advancement toward those goals.

Additionally, the formation and growth of institutions depend on social interaction, collaborative practice, network(s) of actors or agents, and gendered spaces. Our conceptual framework also integrated ideas from the literature on research collaboration, a growing phenomenon across global institutions, to understand the state of collaboration in gender research in the countries where the SGCs are based (Lee and Schottenfeld, 2014). The interaction of SGCs, the formation of partnerships, knowledge generation, and the recognition of a shared problem, such as gender issues in funding groups affect the status of a complex web of institutional policies and resources. Understanding collaboration at the global or structural, national and institutional, and individual (agent) levels is another component to analyzing processes, drivers of change, and impact of gender mainstreaming practices.

## Methodology

The study utilized three approaches: desktop research, focus group discussion and bibliometric analysis to understand the current actions in gender mainstreaming across the 15 SGCs (Table 1) that are part of the SGCI and the status of gender research and collaboration in the participating countries where the SGCs are based. The Institutional Review Board, Office of Regulatory Affairs in the Human Research Protection Program at Michigan State University (MSU) approved the study.

These approaches were used to address the following research questions:

1. What is the status of gender equality and gender policies at the country and institutional levels from the desktop review and the focus group discussion?
2. How is gender integrated into the operations of the SGCs and how does this compare across councils from the focus group discussion?
3. What challenges do councils face in implementing gender-responsive programs supporting STI from the focus group discussion?
4. Is there a potential link between the presence of gender-related programs by SGCs highlighted in the desk review and focus group discussion against the results of the bibliometric analysis focused on the publication output, and collaboration of the SGCs.

### Desktop Research

The desktop review involved secondary data analysis of studies conducted by the African Development Bank (AfDB) and the Southern African Research and Innovation Management Association (SARIMA) about gender and STI in Africa. The gender profiles developed provided insights into the structural, institutional and agent level issues that impact or impede progress toward gender equality.

Three datasets from the SARIMA SGCI project were used, a needs assessment and two benchmarking studies conducted in 2017 and 2018. A total of 191 staff members (111 men and 80 women) at various levels of the organization across all 15 SGCs were interviewed as part of the needs assessment. SGCI focal points at each of the 15 SGCs completed the annual online benchmarking survey to assess their performance and progress in relation to their peers. They provided responses related to the functions of the SGC, gender distribution of employees across the SGC, the type of grants made and the gender of the recipients, the types of institutions that received grants, the countries that they funded, the size of the grants in US$, focus of the SGCs on the SDGs, and the sources of funds for the SGC budget. This data compiled as means and percentages and visualized with graphs, demonstated the institutional and agent level status of gender equality at the participating SGCs.

For systems or country gender profiles, data from the AfDB Gender Equality Index (GEI) was used to compare equality along three dimensions of human development (human economic opportunity, human social development, and human equality in law and institution) from the 15 countries in the SGCI project. The GEI dataset is the most comprehensive assessment of the state of gender equality on the African continent, examining the role of women as producers, economic agents, in human development, and as leaders in public life (African Development Bank, 2015).

### Focus Group Discussion

A focus-group discussion (FGD) with senior level administrative personnel representing each SGC was also conducted to provide perspectives on the institutional actions around gender mainstreaming, gender equality, and STI. A semi-structured FGD with a purposely selected group of 16 individuals representing coordinators and top officials of the 15 SGCs as well as funding partners of the SGCI project was held during the Annual Meeting of SARIMA in Windhoek, Namibia in May 2017. SARIMA was the collaborating technical agency (CTA) contracted for capacity building of the SGCs to manage research. Three academic staff from MSU, one of whom was the project lead for the SARIMA research management (RM) capacity building project of the SGCI, served as facilitators for this focus group discussion. The individuals selected as participants in the FGD were the focal points for the SGCI project and were invited by the executive heads of the SGCs to participate in the FGD as well as attend a research management capacity building program at the SARIMA annual meeting. The participants were provided with information about the FGD including their rights as a research participant to withdraw from the study at any time if required. They signed a consent form to participate. The FGD was held in English and French to accommodate Francophone participants and the session lasted 2 h. The discussion was tape recorded and notes taken by the researchers in the project. The FGD provided data that helped unpack the influence of institutions on gender mainstreaming and gender equality as well as the incentives and capacities of individual agents acting within particular institutional contexts.

### Bibliometric Analysis

Bibliometric analyses was used to investigate the trends in publication outputs and collaboration among SGCI member countries to supplement the discussion on STI indicators, especially on scientific production, uncover any potential impacts that gender mainstreaming might have on gender-related research production, and assess the research performance and collaboration around gender in each of the countries where the SGCs are based.

Bibliometrics has been used extensively to analyze research systems at the global, country and institutional levels and is based on bibliographic information on the enumeration and statistical analysis of scientific output in the form of peer-reviewed journal articles, publications, citations, patents, and other more complex indicators (Goffman and Harmon, 1971; Melin and Persson, 1996; Okubo, 1997; Wagner and Leydesdorff, 2005). Advanced bibliometric methods and visualization techniques (e.g., network maps based on keyword analysis) backed with various forms of analytics has further enhanced the value of bibliometric indicators to present and analyze research activities, emerging research areas, and new clusters of knowledge (Moya-Anegon et al., 2005; Small, 2006; Skupin, 2009; Upham and Small, 2010; Payumo and Sutton, 2015; Payumo et al., 2019). A number of studies have recognized the value of bibliometrics for understanding the link of gender and STI, for example the gender gap in various disciplines such as medicine, and in recognizing gender disparities in research output and collaboration patterns (Soderlund and Madison, 2015; Elsevier, 2017). This type of research offers insights into emerging research areas, fundamental research gaps that might be explored, global collaborations happening and knowledge transfer within the scientific community.

The bibliometric analysis used 10-year publication data (2008–2017) authored and co-authored by scientists in the 15 countries that are part of the SGCI. The data were extracted from Elsevier's Scopus database, the world's largest abstract and citation database of peer-reviewed literature. A basic search strategy was first used to locate gender-related research publications for the SGCI participating countries: article title, abstract, keywords = “gender,” affiliation country = “Africa”; date range = “2008–2017.” A thesaurus containing all topics related to gender research and/or those that has a gender component was also developed to locate more gender-related publications for the SGCI countries. The final search query for the bibliometric analysis then included the basic search strategy and the terms in the thesaurus. An additional filter was then set according to the affiliation country to include only the publications published by the 15 SGCI-participating countries. The final list of keywords is shown in Table 2. Metrics used in this analysis included the publication count of research papers related to gender, the total citation count, the citation per publication (CPP), the collaboration (proxied by co-authorship). The co-authorship network of the SGCI countries was visualized using VoSviewer (van Eck and Waltman, 2018).

**Table 2:** Keywords used to search Scopus for gender-related research publications of SGCI-participating countries.

	**Keywords**
A	Affirmative action
B	Boy(s)
C	Cisgender, cohabitation
D	Domestic violence
E	Emotional labor
F	Family care, family planning, fatherhood, female, femininity/femininities, feminism feminist, feminization (feminisation)
G	Gay, gender, gender-based violence, gendered, gendering, girl(s)
H	Heterosexuality, homophobia, homosexuality
I	Intersectionality, intimate partner violence
L	Lesbian
M	Male(s), marital, marriage, masculinity/masculinities, masculinization (masculinization), maternity, men, misogyny, motherhood
P	Paternity, pregnancy, pregnancy
Q	Queer
R	Rape, reproductive health, rights
S	Sex, sex discrimination, sex trafficking, sex-disaggregated, sex segregation, sexist/sexism Sexual, sexuality/sexualities, stem pipeline/stem equity, structural violence
T	Transgender, transphobia
W	Women, woman

### Study Limitations

There are important caveats to this study. First, we recognize that relying on reports from AfDB and SARIMA only provided us partial answers, either in terms of the precision or the timeliness of the information on the status of gender equality and gender mainstreaming at national and institutional levels. The focus group discussion, which we used to gain in-depth understanding of gender issues and fill this gap, did not explore the nuance of gender identities and the intersections of diverse inequalities as it focused on equality and gender mainstreaming related to women and men, more broadly. However, it could serve as a foundation for future research which could expand the concepts and findings to be inclusive and responsive to other marginalized identities and groups. We also recognize the limitations in the use of bibliometric data, including database coverage, design of search query based on keywords, differences of citation activity between fields of research, e.g., life science vs. social sciences, and that citation is a lagging indicator. The methodology, including the interpretation of the different indicators, however, has been built on best practices in indicators research reported by Moed et al. (2004), Adams (2009), and Rosas et al. (2011).

## Results

### Gender Profile of Selected African Countries and Their Science Granting Councils

**Figure 2:**
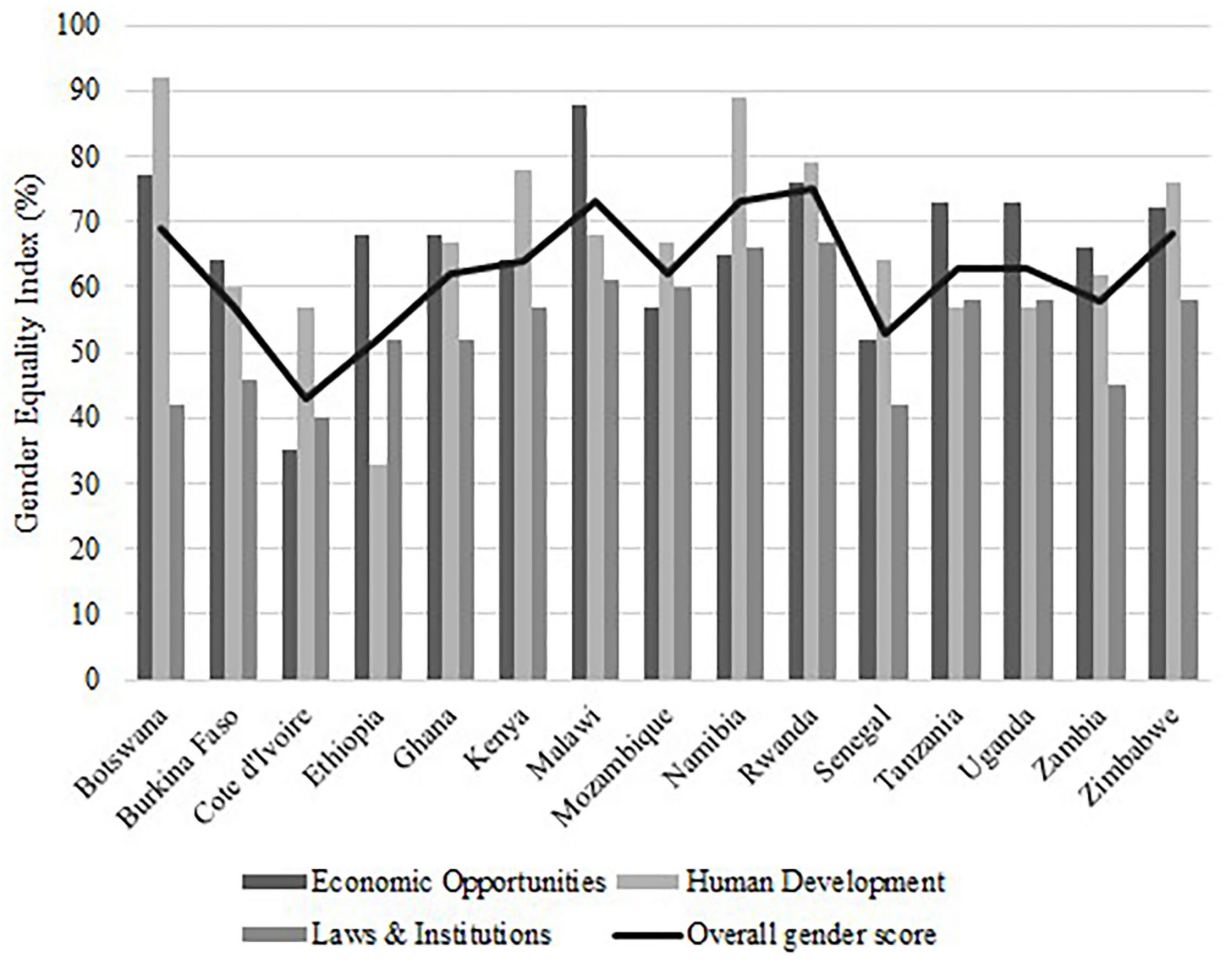
Gender Equality Index of the 15 African countries that are part of the Science Granting Council Initiative (SGCI). The index includes three constitutive dimensions shown by the bar charts: equality in economic opportunities, equality in human development, and equality in laws and institutions. The overall score for gender equality is represented by the solid line. Adapted from African Development Bank (2015).

To understand the underlying structural features which influence gender equality within a particular context, we examined the 2015 Gender Equality Index scores of the 15 African countries that are part of the SGCI. These are shown in Figure 2. The index developed by the African Development Bank (2013) offers a composite measure focused on three important dimensions of gender equality: equality in economic opportunities, equality in human development, and equality in laws and institutions and provides an overall score for gender equality (African Development Bank, 2015). The state of a country's gender equality is reflected on a scale from 0 to 100, where 0 represents the least gender-equal and 100 represents the most gender-equal. Overall, the 15 countries scored between 43 and 75; those in Southern African had higher overall gender equality scores (average of 67) compared with those in East Africa (average of 63) and those in West Africa (average of 54). Malawi had the highest gender equality in economic opportunities (88), Botswana the highest in human development (92) and Rwanda in laws and institutions (67).

**Figure 3:**
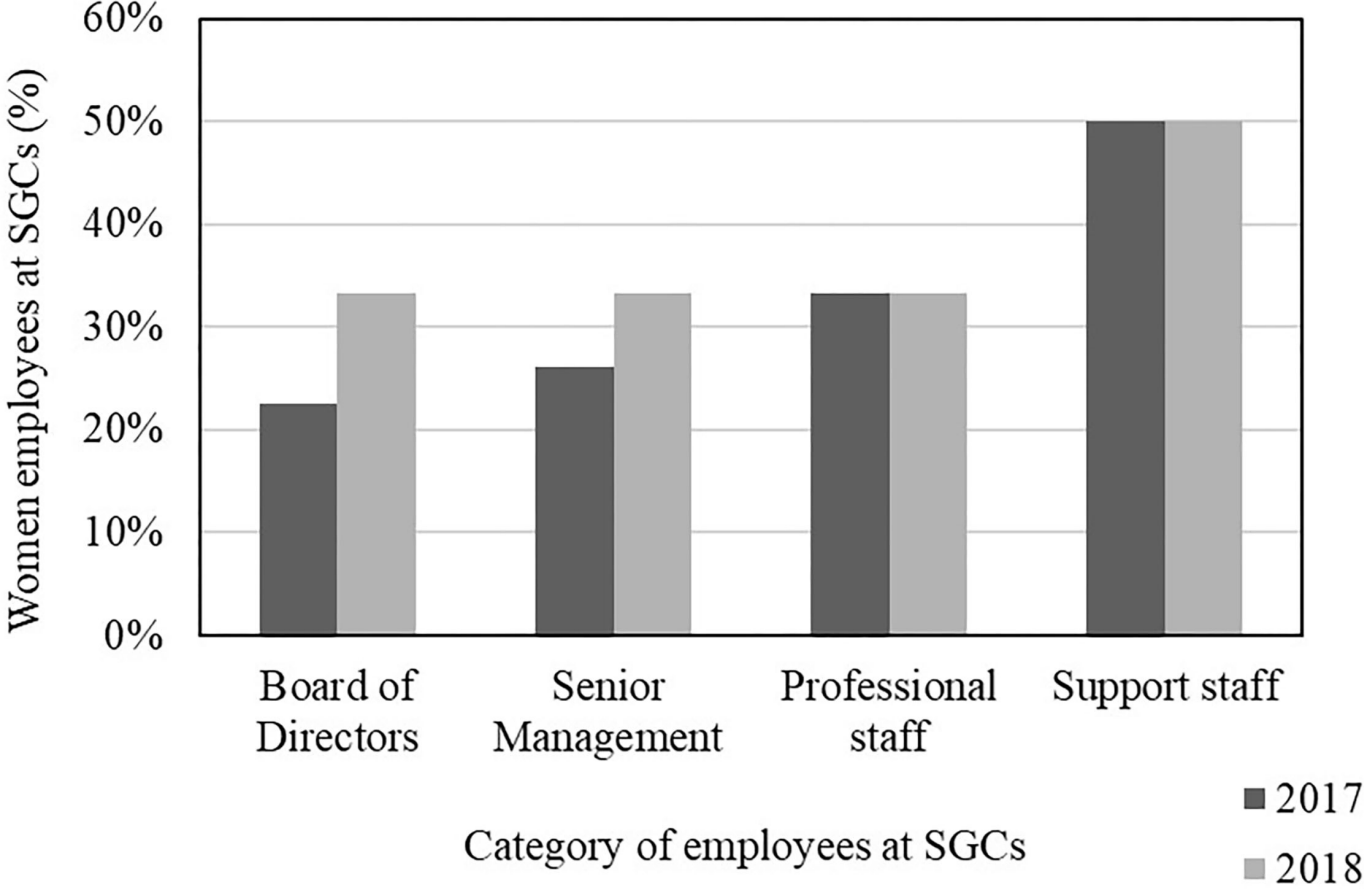
The proportion of women employed or affiliated in selected categories at Science Granting Councils (SGCs) during the period 2017 and 2018. Adapted from Southern African Research and Innovation Management (SARIMA) (2017, 2018).

The gender profile of the staff at the 15 SGCs from the needs assessment and benchmarking study indicate that overall, SGCs employed more men than women (64% men and 36% women). Those in southern Africa had more women overall (42% women) compared to the SGCs in east and west Africa (32% women). The overall proportion of women employed at or affiliated with the SGCs for the period 2017 and 2018 served either as members of the board of directors who were appointed by the relevant government to provide oversight functions at the SGC, management team members, professional staff and/or support staff (Figure 3). For the 2 years, the SGCs had fewer women than men in all categories. In 2017, the board of directors had 23% women and increased to 33% in 2018; senior management had 26% women in 2017 and increased to 33% while professional staff were 33% women in 2017 and did not change in 2018. As expected, support staff had the largest percentage of women, which was 50% in both 2017 and 2018.

**Figure 4:**
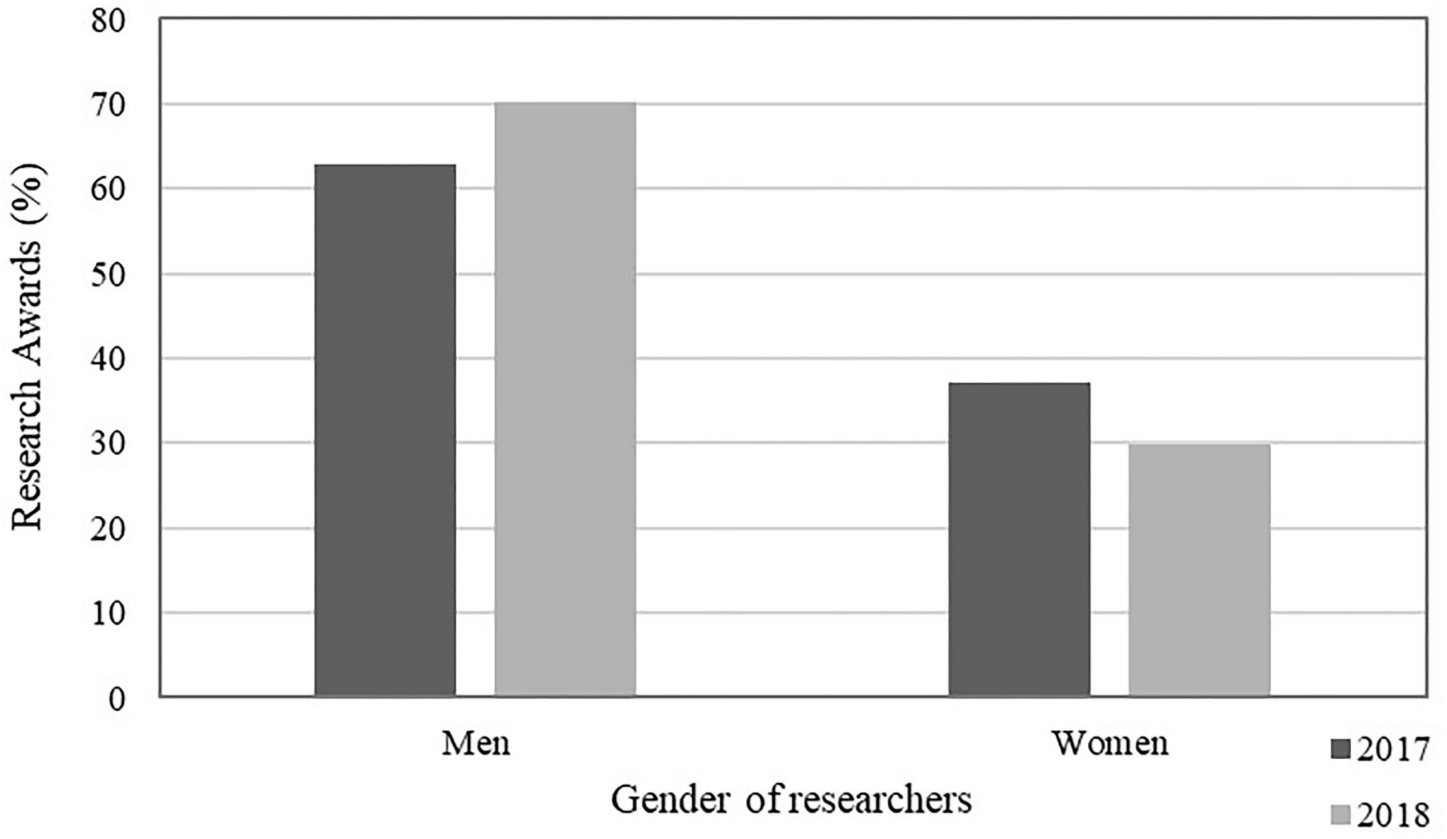
The proportion of research awards from Science Granting Councils (SGCs) that are disbursed to men and women researchers (%) in 2017 and 2018. Adapted from Southern African Research and Innovation Management (SARIMA) (2017, 2018).

The proportion of overall grants awarded by the SGCs according to gender in 2017 and 2018 is shown in Figure 4. More awards overall were given to men (51%) compared to women (49%). Further review of the benchmarking data from the SGCs (not shown in the figure) indicate that for research awards specifically, more were awarded to men (62.8%) compared with women researchers (37.2%). Furthermore, awards of a higher monetary value were more likely to be awarded to men compared to women. SGCs also indicated that the majority of the funding for their programming came from their national government (over 95%) compared with <1% coming from international sources [Southern African Research and Innovation Management (SARIMA), 2017, 2018].

**Figure 5:**
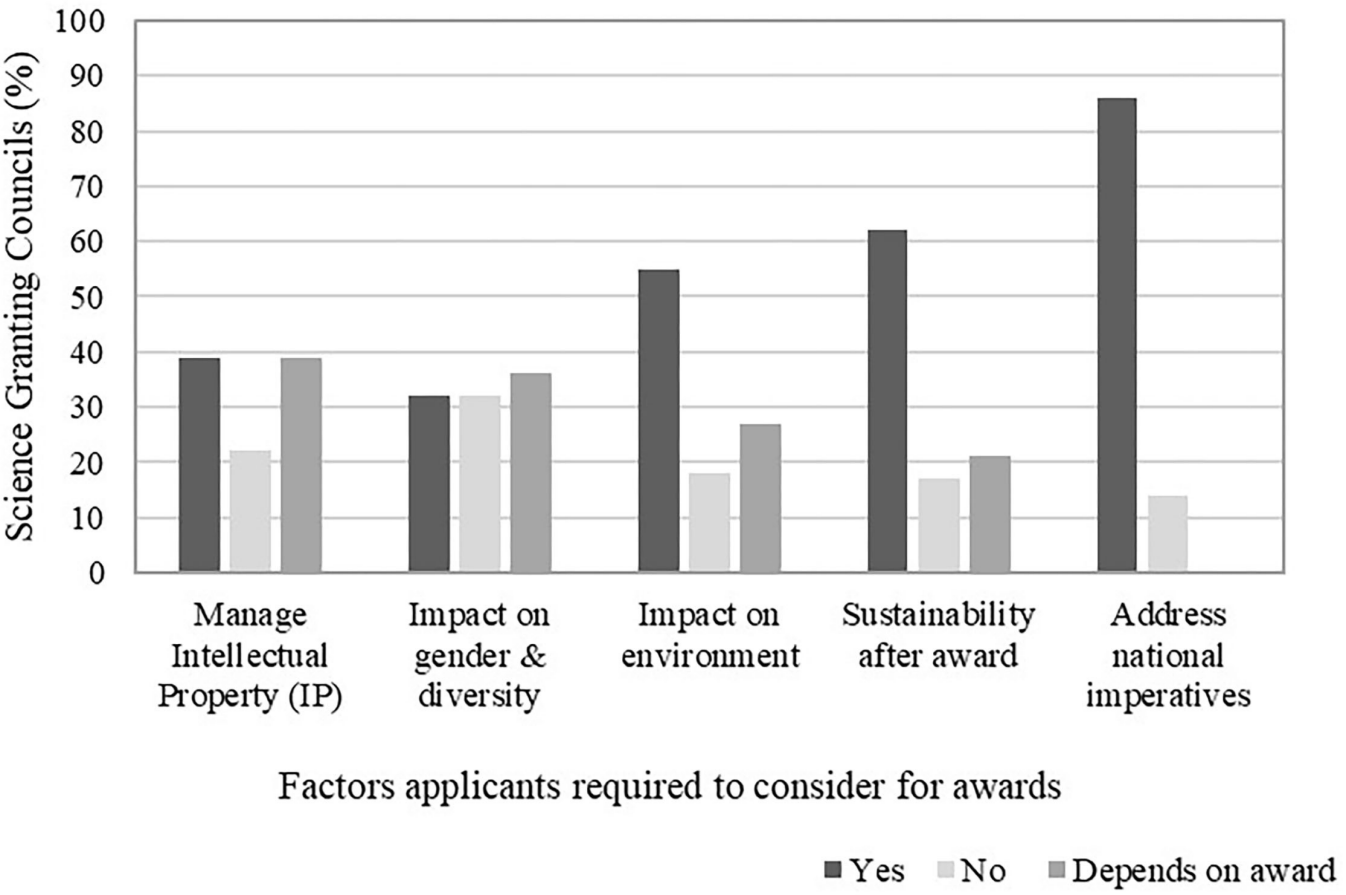
Factors that applicants for research awards are required to consider in their applications to Science Granting Councils (SGCs). Adapted from Southern African Research and Innovation Management (SARIMA) (2017).

**Figure 6:**
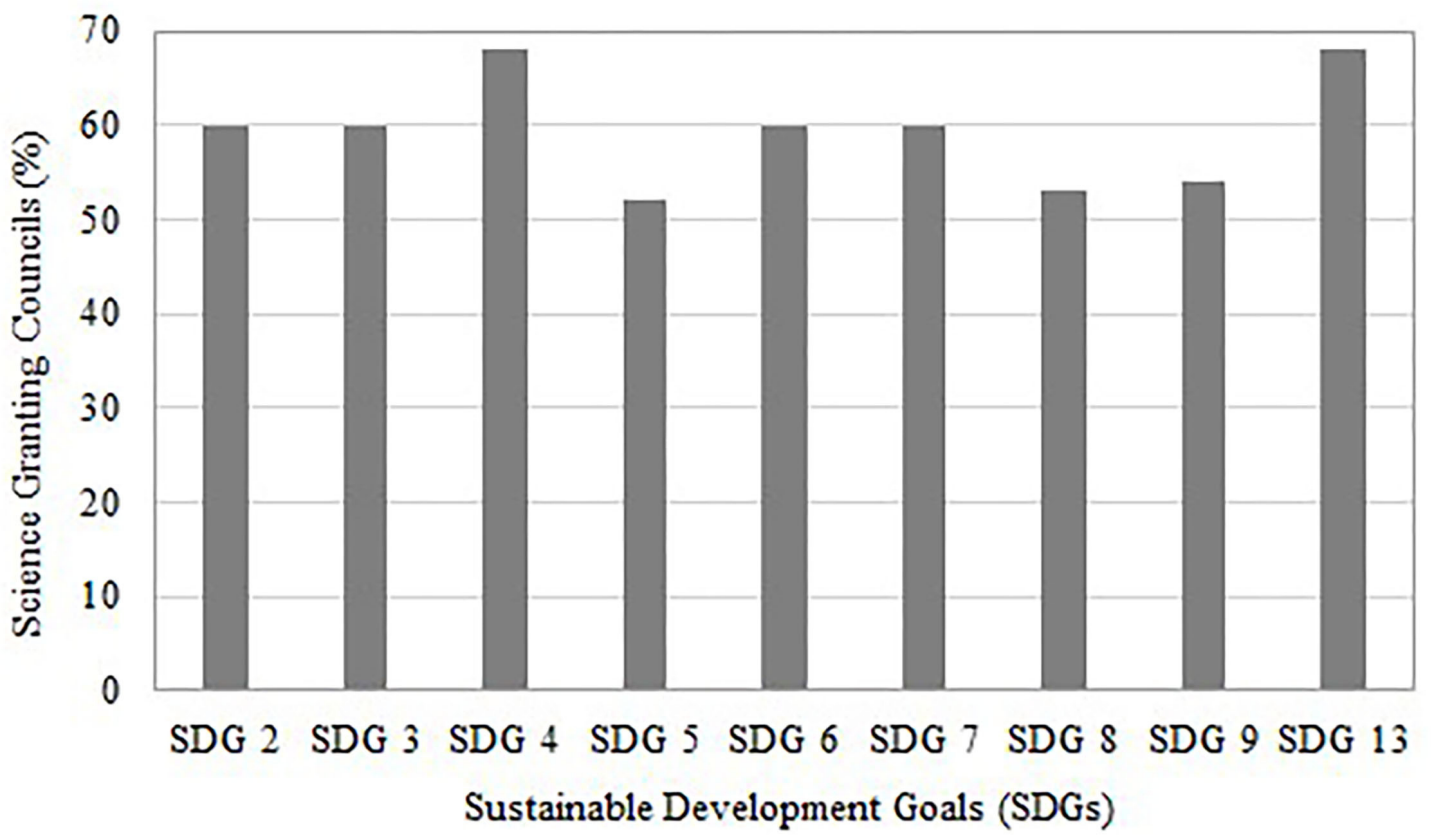
Selected Sustainable Development Goals (SDGs) that were a focus at Science Granting Councils (SGCs) in 2017. Adapted from Southern African Research and Innovation Management (SARIMA) (2017).

The majority of SGCs required applicants to consider the relevance of their work to national imperatives, sustainability of the project after an award as well as the impact on the environment (Figure 5). However, the role of gender and diversity appeared to be less of a priority compared to others as reported by the SGCs in the benchmarking review. This was also further confirmed with the data on the percent of SGCs that focused on selected sustainable development goals (SDGs) (Figure 6); the SGCs placed slightly less emphasis on SDG5 (gender equality), compared to other goals such as zero hunger (SDG2), good health and well-being (SDG3), quality education (SDG4), clean water and sanitation (SDG6), affordable and clean energy (SDG7), decent work and economic growth (SDG8), industry, innovation, and infrastructure (SDG9) as well as climate action (SDG13) [Southern African Research and Innovation Management (SARIMA), 2018].

### Focus Group Results

**Figure 7:**
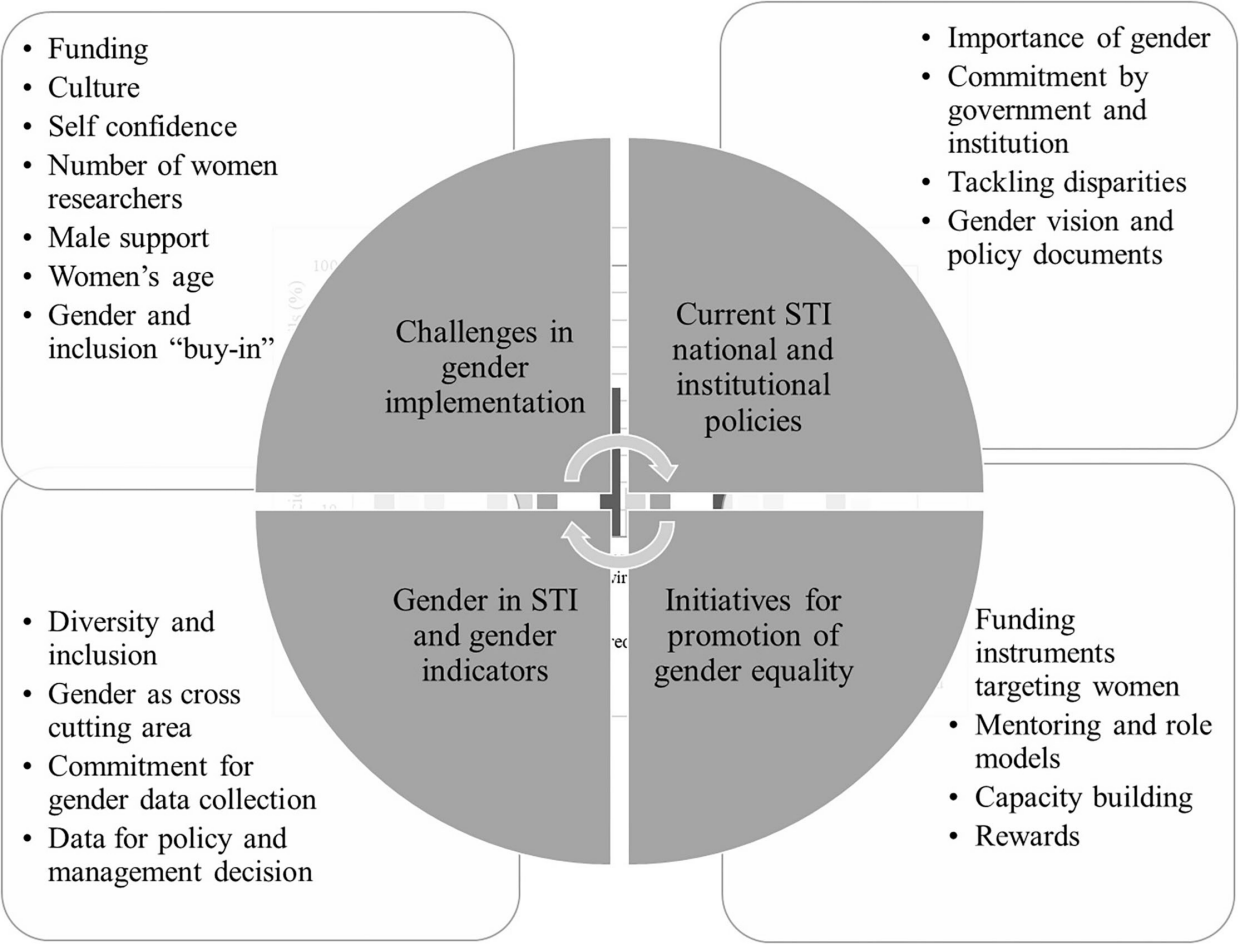
Perspectives of the focal points of the Science Granting Council Initiative (SGCI) on gender and the operations of the Science Granting Councils (SGCs) during the focus group discussions held at the 2017 SARIMA conference.

The views of the participants in the focus group discussion on gender approaches and the process of gender mainstreaming at their SGCs are summarized in Figure 7. These views provide insight into the incentives and capacities for individual agents as well as institutional dynamics for potential change. The discussion focused on the following aspects: (i) existing national and institutional policies on gender and STI, (ii) the current initiatives for promoting gender equality and status of gender mainstreaming approaches in STI, (iii) considerations of gender in STI research and data collection for gender indicators; and (iv) the challenges SGCs face in mainstreaming gender and their role in the promotion of gender equality.

#### Existing National and Institutional Policies on Gender in STI

All representatives of SGCs emphasized the importance of gender and STI to their organizations, although they all recognized that there were differences in the implementation of gender equality policies and practices. They all reported a commitment by their national governments toward tackling gender disparities and mainstreaming gender considerations across all sectoral activities. Many countries have vision documents, national policies, and instruments to promote gender equality, women's desks in various ministries, as well as standalone ministries specifically for gender and/or women's affairs. These policies and other guidance documents were used to guide the gender programming of the SGCs. Only Zambia, Zimbabwe, and Ghana reported having gender mainstreaming initiatives across government ministries. In terms of women's participation at the governmental level, Kenya, Tanzania, and Zambia reportedly had a quota system to ensure that women are represented in parliament. At the time of the focus group discussion, none of the SGCs had a policy or framework to support mainstreaming and promoting gender into STI initiatives. However, some SGCs, including Fundo Nacional de Investigacao (FNI) in Mozambique and the National Research Foundation of Kenya (NRF Kenya), have since developed a policy. These responses on national and institutional commitment to gender indicate that although countries are showing some progress in their commitment to gender, there is still a lot of work to do at the national and institutional levels to effect change especially within the STI space.

#### Current SGC Initiatives for Promotion of Gender Equality in STI

The SGC representatives as part of their role as a catalyst in the STI systems showed commitment toward addressing gender disparities and supporting women scientists and researchers. Indeed, the majority of SGC representatives recognized the importance of promoting gender equality, and some of the councils have implemented specific activities for promoting gender equality in STI. Firstly, they indicated that one of the goals toward promoting the integration of gender in STI is to increase the number of women researchers who are competent in their fields to bridge this gap between men and women in the sciences. A few of the SGCs were designing and implementing gender-specific funding instruments targeted at supporting women researchers for training at masters and doctoral levels. The SGCs in Senegal, Zambia, and Kenya had special funds to promote women's participation in higher education that provided post-graduate scholarship support for deserving scholars. A similar initiative was also being implemented to encourage young girls and boys to participate in science, technology, engineering, and math (STEM) fields at school. In Malawi, the SGC supported an association of women scientists that provided mentoring and role modeling and showcased it on their website to create visibility and to encourage more women into STI. In Ethiopia, the SGC promoted STI capacity building, provided financial support for women scientists in universities, and conducted research workshops to enhance the research and leadership skills of women scientists.

*When it comes to the issues of Science Technology and Innovation, before the inception of NRF Kenya, we had a fund which was run by the government, but it was administered by the national endowment research fund. In that fund, a specific program was supporting women in sciences. The reason being that it was noticed that women were not doing so well in the sciences so it was built to empower them and build their capacity. That program was supporting the research component of women who were pursuing their postgraduate studies. (Kenya)*

*We have two science and technology universities, and we support women scientists with stipends and financial support until they complete their studies. We also organized research methodology workshops and invite successful women who completed their masters and Ph.D. to present on the challenges they had to endure to complete their PhDs and attain professorships and to mentor upcoming women scholars. (Ethiopia)*

SGCs also reported that they focused on mentoring early career scientists and identified senior women scientists as role models to share their lessons learned as well as recommend ways to navigate the career ladder. Additionally, SGCs reported giving research excellence awards in various fields and specifically recognized women's excellence in research by creating a prestigious presidential award dedicated to the best women researcher. One participant indicated that they worked closely with regional economic bodies like the East Africa Science and Technology Commission (EASTECO) to develop regional S&T policies focusing on gender and STI. Despite these initiatives, the SGCs reported that women's participation in STI is still limited in all countries. They were committed, however, to continue to strengthen initiatives for the promotion of gender equality in STI.

*We have a female post graduate scholarship fund that supports women researchers who want to do masters and PhD studies and it is restricted to women based at universities in the country. (Zambia)*

*We made deliberate efforts that one of the most prestigious awards – the presidential award is given to a woman researcher. (Zimbabwe)*

#### Considerations of Gender in STI Research and Collection of Gender Indicators

SGC representatives demonstrated a recognition of the necessity of diversity and inclusion for research excellence and the need to involve women in research and research management by the SGCs. They all agreed that research affecting women should not be conducted without involving women and similarly for research on men's issues. Therefore, it was recognized that this requirement should be factored into calls for proposals and that the research management staff at the SGCs needed to be made aware of this requirement.

*You cannot do women's health research without women involvement in the research team. In the same way, you cannot do male health research without men. Some of the challenges are not at the higher level but in the specific program development level and must be factored into the call for proposals. (Kenya)*

The representatives highlighted that their SGCs are committed to monitoring gender progress and collecting gender-disaggregated STI data, which they did in their funding applications. The SGC in Ghana, for example, indicated that gender inclusiveness was recently highlighted in their national research and development (R&D) survey and used that data to justify a request for additional financial support from the national government.

*For our SGC, the initial R&D survey we did generated information about the real picture on R&D expenditure. This influenced the government to increase funding from $2.5 million to 30 million. The increase in funding represents a very big jump. I think it (R&D survey) is a useful initiative and tool that will enable SGCs to design evidence-based programs. (Ghana)*

Participants agreed that SGCs must be able to attach meaning to the data collected and provide timely advice to policymakers for decision-making. Using STI indicators for policy and decision-making, however, was still a challenge in many countries as it was still new in the African context, and even though it is improving, participants acknowledged that it could be used more robustly. Many of the SGCs noted that the data they collected could be used for transforming the STI system, for example, that increasing the numbers of empowered women scientists contributes to the socio-economic transformation of the country. They asserted this should be explained to policymakers. The SGCs noted that one of the objectives of the SGCI is addressing this gap and strengthening the capacity of the SGCs as champions of indicators in public policymaking in general. It was expected that their role in using and translating gender-specific indicators would also be strengthened in the future.

*I also think that data collected should be linked to developmental goals. For example: the question can be asked that even if we have the numbers (of women researchers), what contributions have they made. These numbers should be tied to the developmental agenda. Have the numbers of women researchers empowered contributed to the socio-economic transformations? (Ghana)*

*The issue of indicators is still new in this context. It is improving, and it can be used in more robust ways. We should be able to understand what the numbers mean. If there are so many woman, then what? We have to be able to attach more meaning to the numbers. (Kenya)*

#### Challenges in the Implementation of Their Role and Promotion of Gender Equality

The SGCs that stated that they have dedicated gender-related programming that targeted women researchers included PASRES in Côte d'Ivoire, NRF Kenya, FNI of Mozambique, NCST in Malawi, and DFRDT in Senegal. Although many of the other SGCs did not have dedicated programs, those like the NSTC of Zambia and MoST in Ethiopia had offered workshops to build capacity of women researchers in grant proposal writing.

The majority of SGCs admitted that while efforts are made to promote gender equality and commitments have been made by the SGCs, the progress has been slow. Institutional norms and culture were discussed as barriers for the involvement of women in STI; however, this received mixed reviews as some of the SGCs felt for example, that there was now an over-emphasis on culture. It should be noted, however, that researchers have reported on the patriarchal systems in which both cultural and religious values that define the position and role of the sexes often presents challenges to gender equality (Bassey and Bubu, 2019). Some SGCs acknowledged specific examples of marriage and children as cultural issues that impact women's decisions and career advancement. However, they concluded that culture itself is dynamic, and there was a need for in-depth studies to review why there is still a disproportionately low number of women in STI, rather than simply attributing it to culture.

*As an SGC, we have taken that step to recognize female scientists and emphasis is placed on the female scientists. The way we understand our role as SGCs is that we are a catalyst with STI systems. We link government, knowledge generators (universities), funders of research and users of that knowledge. Our role is to facilitate linkages within this ecosystem. We try to perform our role to the best of our abilities but there are challenges. Sometimes our role is not fully understood by the actors in these ecosystems. (Zambia)*

One of the other major challenges observed by the SGCs is that women scientists seem to lack self-confidence and belief in themselves and do not perceive that their male colleagues are supportive. They cited as an example, the low numbers of proposals received from women scientists when they issue calls for proposals. In some cases, only one woman scientist would apply for a given call for proposals. These two points seemed to be related and potentially formed a vicious cycle for women's research leadership. Women scientists seemed to lack the confidence to apply, thinking that they are not qualified or that they will not be successful, hence, ended up not applying resulting in low numbers of applications from women.

*The challenge in Zimbabwe is that even when we come up with these initiatives like for calls, the female turn out is very negligible. Even the nomination for awards, you have very few women. The reason why women are not forthcoming might have to do with those structural issues that are there for quite some time. (Zimbabwe)*

*In Zambia, the challenge is the lack of applicants. We issue the call but sometimes it is only one woman who apply. (Zambia)* *There are those men who also resist women's participation and do not want to see women excel. There is a need to engage them with capacity building. (Kenya)*

*The other problem is women themselves exaggerate the gender differences. Rather than see their counterparts as supportive, they see us as antagonist. But it is also the same with some men who think they are superior to women. Therefore, we must work on the attitudes and culture*. (*Ethiopia)*

They also highlighted a lack of opportunities in the sciences for women and restrictive policies by funders for women. For example, the age of women applicants can sometimes work against them. In many cases women's professional careers are delayed due to family commitments, and they often do not qualify under age-restricted eligibility requirements. The age barrier is increasingly being addressed by many funders, but it still came up as an issue. Some SGCs also acknowledged having funding challenges as highlighted by participants from Zimbabwe and Kenya. One of the roles that SGCs play is providing funding to institutions, individual researchers, and postgraduate students, and in many cases, they were struggling to fulfill that mandate due to limited government funding and limited engagement with other funders for joint calls for proposals.

There was some discussion amongst the group about whether gender and the importance of inclusivity were understood or wanted. Some SGCs felt that work in this area was driven by applicants' desire to get a grant approved and by international policies and resolutions such as the SDGs instead of a “bottom-up” interest and desire. They further explained that for many it was simply about “adding women” to a research team or as participants in a program and that many funders have played into this narrative. In many cases, applicants simply ticked off boxes in terms of numbers, believing that they have considered gender. Although participants recognized that including women as participants was important for the SGCs, they acknowledged that more needed to be done for a more transformative impact.

*In most of our template as funders, there is a component on gender and so people just complete it to fulfill the criteria without an understanding. They feel that it is something they need to do to get the project. It is kind of a supply driven issue—you just must show something that you have gender. (Kenya)*

Participants recommended that SGCs introduce empowering and capacity-building programmes focused on issues such as implicit bias that would address attitudes, change mindsets and boost women's confidence to know that they can be just as successful as their male counterparts. They added that men also needed to be part of the capacity building as some men resisted women's participation and did not want to see women excel. There were also recommendations that SGCs should directly and/or advocate for research institutions to provide support like family leave opportunties, childcare, pregnant and nursing centers at workplaces, hence, encouraging participation in research.

### Bibliometric Results

#### Publication Outputs and Citation

**Figure 8:**
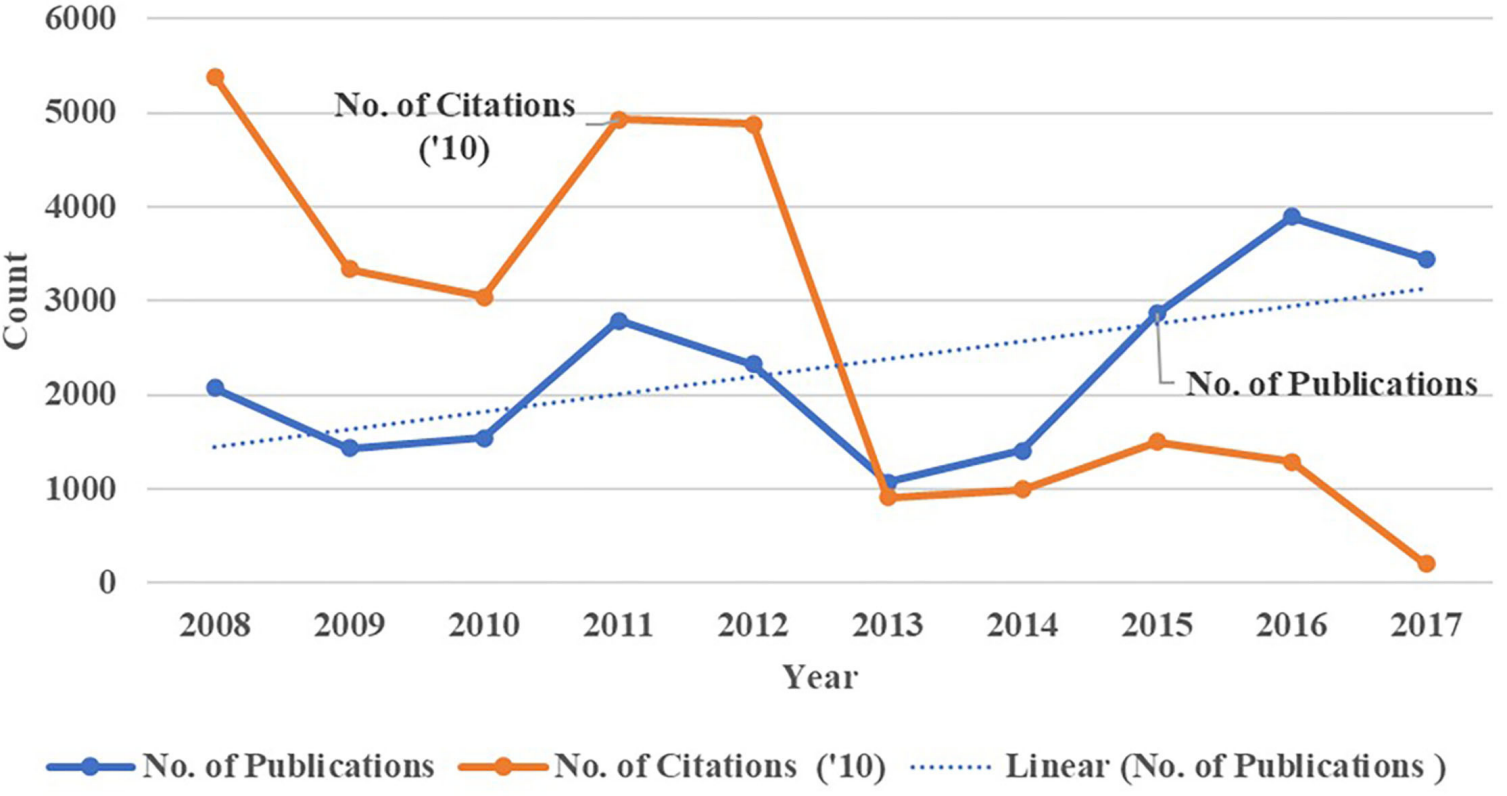
Growth in the overall number of gender-related publications and citations for SGCI participating countries, 2008–2017. Source: Scopus.

The 15 SGCI participating countries produced gender-related research outputs covering the period 2008–2017, which are presented in Figure 8. An increased publication output was noted with more than 22,842 gender-related papers and an annual output increasing 6% per year. The highest publication volume was in 2016 while the lowest publication count was in 2013. The gender literature produced by researchers from the 15 SGCI participating countries appeared in different types of publication sources, with most of them published as articles in journals. A steady upward trend on gender-related research outputs was observed for the 15 SGCI participating countries and when analyzed individually as shown in Table 3. Ethiopia, Kenya, Ghana, Uganda, and Tanzania were the top five contributors of gender-related papers published during the 2008–2017 period, while Namibia contributed the fewest. The full data supporting the bibliometric analysis, which can be used for additional data analysis and visualization is available via: https://doi.org/10.7910/DVN/0QEFTQ.

**Table 3:** Comparison of 2008 and 2017 publication output, citation, and CPP SGCI participating countries (sorted alphabetically by country name).

**SGCI country**	**Publication output**		**Citation**		**CPP**	
	**2008**	**2017**	**2008**	**2017**	**2008**	**2017**
All countries	2100	3630	53815	1947	26	1
BWA	6	145	177	118	30	1
BFA	106	164	2201	71	21	0
CIV	112	98	1979	55	18	1
ETH	218	757	4346	390	20	1
GHA	171	512	4749	373	28	1
KEN	501	656	13629	459	27	1
MWI	145	238	4021	220	28	1
MOZ	57	100	1270	69	22	1
NAM	20	48	212	23	11	0
RWA	31	93	1033	105	33	1
SEN	143	180	2287	87	16	0
TZA	248	394	7367	345	30	1
UGA	291	540	10836	381	37	1
ZMB	125	177	4634	144	37	1
ZWE	102	172	3459	110	34	1

Citation counts indicate that the gender-related research outputs by the SGCI countries are being used by their colleagues in their research. The citations from 2008 to 2017 for the gender-related publications totaled 264,000 with an average CPP at 12. Citation to these publications was highest in 2008 and lowest in 2017. Botswana generated the highest growth rate in publication output at a 42% compound annual growth rate (CAGR) (Table 3). Côte d'Ivoire, on the other hand, had a negative publications growth rate during the 10-year period, which could have affected its citation volume. Mozambique had the highest CPP while Ethiopia had the least CPP.

Keywords provided by authors of the publication output for more than 20 times were analyzed in the supplemental analysis (see Supplementary Figure 1). More than 5,726 keywords met the threshold that resulted in the following clusters: (1) gender attitudes, (2) women in agriculture; (3) sex and gender in African vaccine development; (4) gender disparities in healthcare; (5) gender and nutrition; and (6) gender-based violence and HIV/AIDS.

#### Collaboration

**Figure 9:**
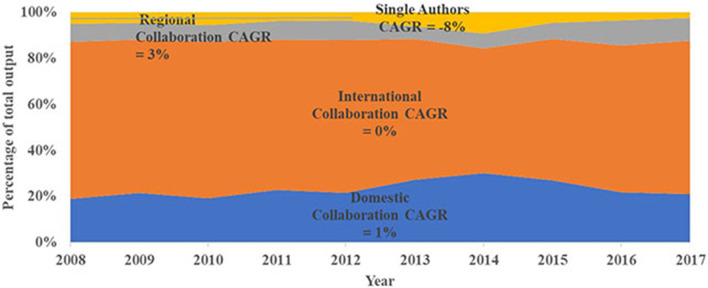
Percentage distribution of different types of collaboration for countries participating in the Science Granting Council Initiative (SGCI) based on co-authored publications related to gender by year for the period 2008–2017. Single authorship was included in this graph for illustration purposes. Source: Scopus.

Gender-related research for SGCI participating countries involved co-authorship and collaboration, which was consistent throughout the years 2008–2017 (Figure 9). More than 95 percent of overall gender-related publications for SGCI participating countries involved multiple authors and institutions. The majority (65%) of this collaboration was due to international collaboration, followed by domestic collaboration (22%), and regional collaboration (8%). Regional collaboration showed the highest growth rate at a CAGR of 3 percent. On the other hand, single authorship continued to decrease at a rate of 8 percent per year. These results confirm the observation that gender research in SGCI participating countries is increasingly conducted by groups of collaborating researchers rather than by single researchers—a phenomenon also observed in other research areas (Glanzel, 2001, 2002; Glanzel and Schubert, 2004; Koseoglu, 2016). This collaborative culture is also increasingly encouraged, especially in research areas that address scientific questions dealing with global challenges (Stvilia et al., 2011).

**Figure 10:**
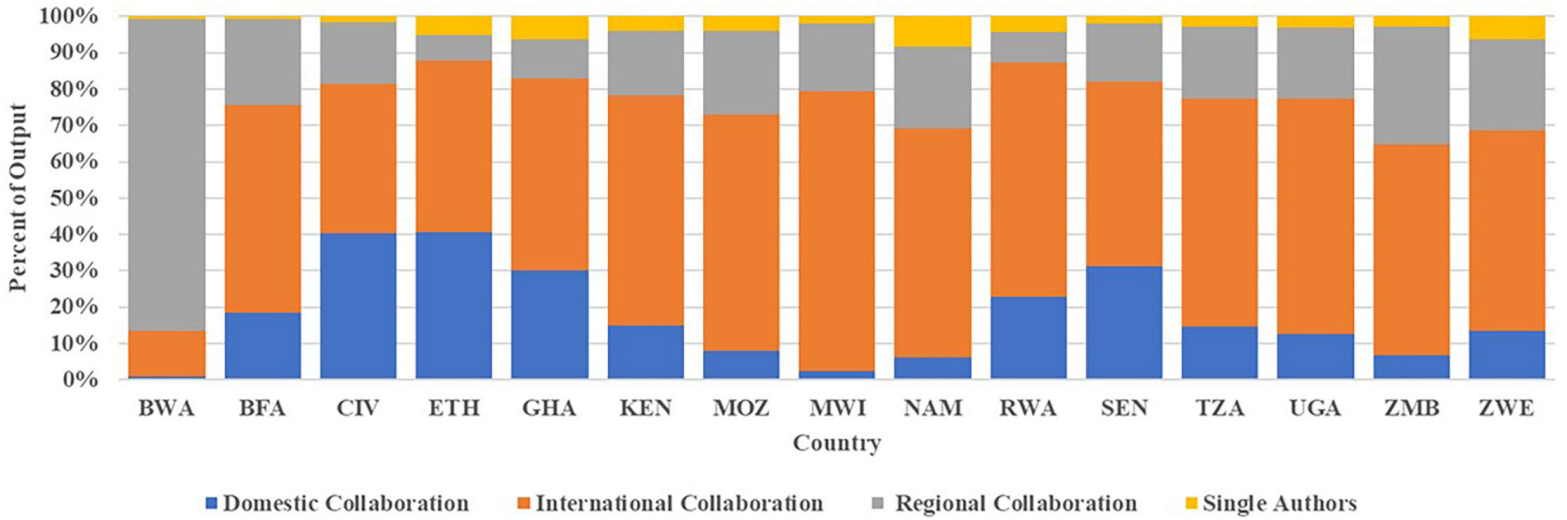
Percentage distribution of different types of collaboration for countries participating in the Science Granting Council Initiative (SGCI) based on co-authored publications related to gender by country for the period 2008–2017. Single authorship was included in this graph for illustration purposes. Source: Scopus.

**Figure 11:**
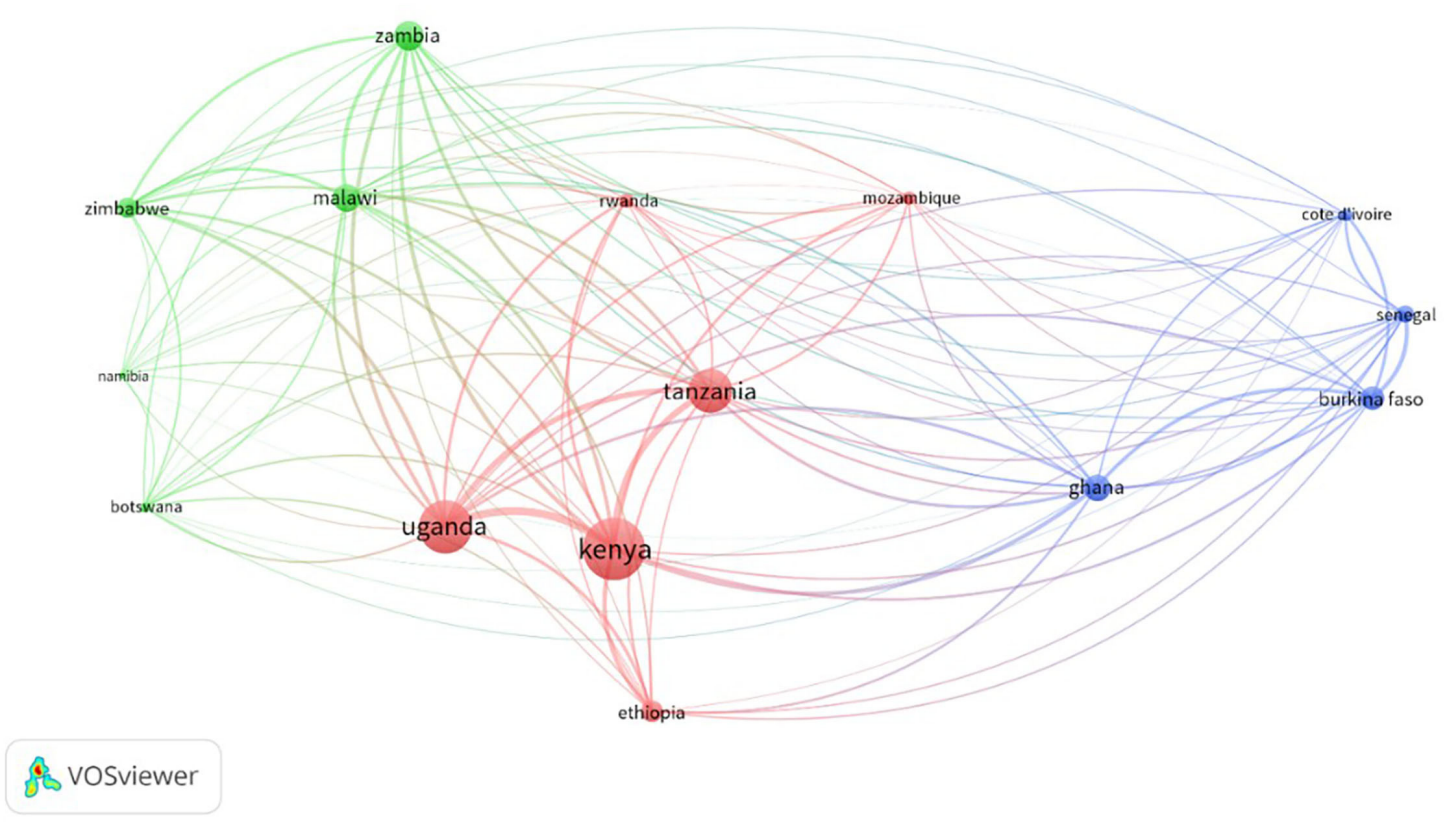
Network visualization of the regional partnership in gender-related research between the 15 countries participating in the Science Granting Council Initiative (SGCI). Each circle, or node, in this network, represents an SGCI-member country, and lines between circles represent co-authorship. The size of the circle corresponds to the number of articles published by that country within our dataset. The thickness of the line represents the frequency of the co-authoring.

As expected, gender publications that involved international partnerships received the highest citation count (*n* = 189,540) whereas publications that involved single authorship received the lowest citation count (*n* = 2,988). These results support the observations of Katz and Hicks (1997) and Rigby (2009) that international collaborations tend to produce more highly cited papers than collaborations of experts in a single country. This observation is also true for regional collaboration. Figure 10 presents the different types of collaborations for individual SGCI participating countries. The growing internationalization of gender research is noted for most of the SGCI participating countries with only three countries (Botswana, Côte d'Ivoire, and Ethiopia) having <50% internationally co-authored publications. Of the 15 countries, Malawi had the highest percentage (77%) for international collaboration while Botswana had the least (13%). Although Botswana published least with international authors, it is the most active in terms of regional collaboration with 86% of its gender-related publications authored with researchers from other SGCI countries. All other SGCI participating countries have records of co-authored publications with another SGCI country. Domestic researchers authored more than 40% of the gender-related publications of Ethiopia and Côte d'Ivoire. Figure 11 is a network map showing the regional partnership among the SGCI participating countries and suggests that collaboration was more common in countries that are close to each other and in the same region. The cluster maps generated from the VoSViewer programme revealed a coalescence of countries belonging to the same region: West Africa, Southern Africa, and East Africa.

## Discussion and Conclusions

Overall, findings from this mixed-methods study indicate that gender-related initiatives in the SGCs are still in their infancy, but they are gaining some traction. The top five gender-equal countries that were part of the SGCI according to the AfDB gender equality index are countries in East and Southern Africa and include Rwanda, Namibia, Malawi, Botswana, and Zimbabwe. This suggests that these countries are making steady steps to improve gender equality, but others mostly in West and East Africa like Côte d'Ivoire, Ethiopia, and Senegal still have some way to go. The index should be able to guide countries in priority policy and program development and implementation to address some of the most serious barriers that prevent, for instance, African women scientists from engaging on a level playing field with men and achieving gender equality (SDG5). For example, each of the five countries scoring lowest in the GEI, Côte d'Ivoire, Senegal, Mozambique, Burkina Faso and Kenya could prioritize the gender gap in salaries at research organizations. The SGCs in Ethiopia, Côte d'Ivoire, Tanzania, Uganda and Burkina Faso may need to prioritize women in terms of training at the higher education level. Finally, the SGCs in Côte d'Ivoire, Senegal, Botswana, Zambia and Burkina Faso could prioritize support for policy and program development to prevent sexual harassment and gender-based violence at research organizations and greater representation of women on the boards of directors and management to advocate for gender equality. These longer-term policy and program initiatives remain a work in progress and many of the SGCs and their countries overall, need to continue to advance their competency in these areas and lay out some concrete action plans toward achieving SDG5.

Results of the focus group discussion highlighted the importance and value being placed on mainstreaming and integrating gender issues across the 15 participating SGC countries in Africa both in terms of promoting gender equality in STI and inclusion of gender-related content and analysis in research. Comparing across the institutions, three common objectives for gender mainstreaming emerged. The first objective was to increase the number of women in STI and promote the full use of human capital to contribute to higher research performance, including authorship and collaborations. The second objective was to increase the number of women in leadership positions in STI research, policymaking, and policy implementation. This goal aimed at a competitive global STI economy through having women scientists—their experiences, perspectives, and expertise—in decision-making positions. The third objective was focused on integrating gender dimensions in research content and curricula, which implied considering the inclusive biological, social, and cultural characteristics of sex and gender throughout the research, teaching and curriculum development process to address the needs of women and girls as well as boys and men, and to make STI attractive to both women and men [Schiebinger and Schraudner, 2011; Tannenbaum et al., 2016; European Commission (EC), 2019]. Some SGCI members are setting the pace in terms of investment to promote and monitor the success of gender mainstreaming and gender equality initiatives through various STI indicators. However, successes and milestones in this area have yet to be achieved in a few of the SGCs, especially those with no clear policies for instituting gender measures and mainstreaming gender into the organizations and their programs.

This finding concurs with a recent scoping study supported by IDRC in 17 sub-Saharan African countries, which highlighted, among many things, the interrelated challenges facing the councils (International Development Research Center (IDRC), 2018). Although that study was not directly related to gender mainstreaming, it brought to light challenges that also hinder gender mainstreaming efforts. These include limited capacity, inadequate funding, overlapping roles, poor coordination with other agencies, lack of appropriate legislation, and poor implementation of science and research funding policies. All of these observations are understandable, especially, since these SGCs are located in countries that are developing their research systems efforts, and with differences in national institutional arrangements at the levels of the STI systems, education and training, and finance. Institutional systems and subsystems as noted by Lundvall and Lema (2014) will impact how new knowledge and information are developed, used, and institutionalized within organizations and these organizational differences will influence the pace (fast or slow) and style of implementation.

The results of the focus group discussion point to the need for a more in-depth understanding of the barriers to women in STI in Africa and the role of confidence-building, mentoring, and role modeling as tools for advancing women in STI. Also, it demonstrated a need for greater understanding of STI indicators as they relate to gender and the relevance of gender data for insights and decision-making. The use of STI indicators, such as those supporting research and scientific productivity, is one of the overall objectives of the SGCI and will require more support in terms of human and institutional capacity building, especially in quantitative science and technology research. This study provided some information and approaches to understanding the gender research landscape in these countries, and a similar approach can also be used by SGCs to map scientific outputs, including their impact in other critical research areas. IDRC issued a call for proposals on *Breaking Systemic Barriers to Women's participation in Science* in 2019; five of the 11 proposals supported were from Africa and are currently in progress. In addition, the SGCI in partnership with the Organization of Women in Science for the Developing World (OWSD) South African National Chapter, and the German Research Foundation (DFG) also issued a call for proposals in 2019 to understand the broader concepts of intersectionality in research as a way to further improve STI systems.

Our results, although only focused on gender-related research for SGCI participating countries, validate findings by New Partnership for Africa's Development (NEPAD) (2014), which reported improvement in the growth rate of scientific production of member countries of the African Union. The increased interest in gender-related research by the 15 SGCI participating countries, however, contrasts with what is happening in the Western World. The study by Joshi et al. (2015) showed that a sharp decline in the frequency of articles published on the topic of gender was observed since the 1980s pointing to “gender fatigue” and even weariness with gender research.

The contribution of research output in the majority of African countries compared to the world output remained very low (New Partnership for Africa's Development (NEPAD), 2014). However, our analysis of the 10-year publication output of SGC participating countries in gender research provided some evidence of progress. The bibliometric assessment showed the positive relationship between the gender-related programming of the SGCs and the level of gender-related outputs and collaboration in the countries where they were based, indicating a potential link between gender mainstreaming efforts and an increase in gender-focused research outputs. This relationship can be validated with additional correlation analysis in the future. For now, we recommend that there should be increased use of bibliometrics to map the state of publication gaps and identify potential gaps in gender-related research and encourage increased publication and citation behaviors among African scientists. Overall, despite some of its limitations, the bibliometric analysis provided a wealth of information that when used with other STI indicators can help reveal the trends and developments in STI research, such as gender-related research. Regular monitoring and evaluation of outputs and impact of gender-related research and/or gender disparity using bibliometrics guided with an expanded thesaurus of this evolving interdisciplinary field are encouraged to help shape policies for additional support and facilitate knowledge sharing and experiences in this area. Likewise, a study that will help link the increased gender-related scholarly activity and its contribution to STI advancement and economic transformation is a worthwhile activity.

Collaboration is widely regarded as beneficial to science in many ways (Katz and Hicks, 1997; Glanzel and Schubert, 2004). The results of the bibliometric study on collaboration networks among the SGC participating countries show the growing internationalization of research, with an encouraging growth rate. The increasing number of collaborative research teams in gender-related publications is also an encouraging result. These increases suggest an increase in the pool of researchers and a change in the balance of research focused more on collaborative teams among African researchers and their partners and not on lone scientists. This upward trend in multi-authorship is expected to continue. However, the slow increase in regional partnerships in gender research was also noted. Information about patterns of collaboration in the SGC participating countries indicates that scientists within Africa rarely collaborate with each other but seek more international partnerships outside of Africa [New Partnership for Africa's Development (NEPAD), 2014]. However, collaborations whether at regional or global levels, remain important enablers to advancing gender-related research objectives and sharing knowledge with the scientific community. Regional partnerships, or among SGCI participating countries are still limited, although the increasing trend is also encouraging. It remains to be seen whether regional collaboration on gender-related research and whether women's participation in these collaborative networks will continue to expand. Again, regular monitoring and evaluation of these partnerships will be very important to assess their impact on gender, STI and SDG5.

This paper adds to a growing body of knowledge that shows the important role that the science granting councils in Africa play in advancing STI, gender equality and achieving SDG5. It highlights some of the gaps in actions, the progress that have been made at country and institutional level, and points to some of the areas where more work is needed.

Since the completion of this study, the funders of the SGCI had an open call for proposals to offer training and technical support to SGCs (and related organizations such as commissions or funds) in specific areas of gender and inclusivity in STI namely: promoting (i) the equality and status of women in research environments, (ii) diversity and inclusivity beyond gender equality in research environments; and (iii) the sex, gender, and inclusivity dimension in research design and content. This is a positive step that demonstrates the commitment that is being given to gender and inclusivity in the SGCI. A follow-up study should be conducted in the future to assess the specific impact of this new funding program on the mainstreaming and integration of gender in STI at the SGCs and in implementing SDG5.

## Data Availability Statement

The original contributions presented in the study are included in the article/Supplementary Material, further inquiries can be directed to the corresponding authors.

## Ethics Statement

The studies involving human participants were reviewed and approved by the Institutional Review Board, Office of Regulatory Affairs in the Human Research Protection Program at Michigan State University (MSU). The patients/participants provided their written informed consent to participate in this study.

## Author Contributions

JJ, JP, and AJ conceptualized the research, participated in the data collection and analysis, interpretation of data, and manuscript writing. MC transcribed the data and participated in manuscript writing. PC participated in the literature review and participated in manuscript writing. All authors approved the version to be published, and agree to be accountable for all aspects of the work in ensuring that questions related to the accuracy or integrity of any part of the work are appropriately investigated and resolved and whose names appear on the submission made substantial contributions to the manuscript.

## Funding

This work was supported by the International Development Research Council of Canada (IDRC), the National Research Foundation of South Africa (NRF), and the Department of International Development of the UK (DFID) that funded Phase 1 of the Science Granting Council Initiative of Sub-Saharan Africa. The research team acknowledges the Centre for Research on Evaluation, Science and Technology (CREST) at Stellenbosch University, the Association of Commonwealth Universities (ACU), the East African Research and Innovation Management association (EARIMA), West African Research and Innovation Management Association (WARIMA), and the Central African Research and Innovation Management Association (CARIMA), who were consortium partners with the Southern African Research and Innovation Management Association (SARIMA) and contributed to the data collection for objective 1 in the SGCI project.

## Conflict of Interest

The authors declare that the research was conducted in the absence of any commercial or financial relationships that could be construed as a potential conflict of interest.

## Publisher's Note

All claims expressed in this article are solely those of the authors and do not necessarily represent those of their affiliated organizations, or those of the publisher, the editors and the reviewers. Any product that may be evaluated in this article, or claim that may be made by its manufacturer, is not guaranteed or endorsed by the publisher.

## Supplementary Material

Supplementary Figure 1 Bibliometric analysis of the keywords in gender-related publications of SGCI-member countries. The size of nodes indicates the frequency of occurrence. The curves between the nodes represent their co-occurrence in the same publication. The shorter the distance between two nodes, the larger the number of co-occurrences of the two keywords.

## References

[B1] Adams J. (2009). The use of bibliometrics to measure research quality in UK higher education institutions. Arch. Immunol. Ther. Exp. 57, 19–32. 10.1007/s00005-009-0003-3 19219531

[B2] Adefuye A.O. Coetzee L. Janse van Vuuren C. Busari J.O. (2020). Medical educators' perceptions of research culture in a faculty of health sciences: a South African study. Teach. Learn. Med. 33, 509–24. 10.1080/10401334.2020.1847653 33272044

[B3] African Development Bank (2013). (AfDB) The State of Gender Equality in Africa: Trends, Challenges and Opportunities. AfDB, Abidjan, Côte d'Ivoire.

[B4] African Development Bank (2015). (AfDB) empowering african women: an agenda for action - Africa gender equality index 2015. gender and social development monitoring division, quality assurance and results department. Afr. Dev. Bank Group. Côte d'Ivoire. Available online at: https://www.afdb.org/fileadmin/uploads/afdb/Documents/Publications/African_Gender_Equality_Index_2015-EN.pdf (acessed: October 10, 2021).

[B5] African Union Commission (AU). (2014). Science, Technology and Innovation Strategy for Africa 2024. Available online at: https://au.int/sites/default/files/newsevents/workingdocuments/33178-wd-stisa-english-final.pdf

[B6] African Union Commission (AU). (2015). Agenda 2063: The Africa We Want. Available online at: https://au.int/sites/default/files/documents/33126-doc-framework_document_book.pdf

[B7] Airini S.C. Conner L. Mcpherson K. Midson B. Wilson C. (2011). Learning to be leaders in higher education: what helps or hinders women's advancement as leaders in universities. Educ. Manage. Adm. Leadersh. 39, 44–62. 10.1177/1741143210383896

[B8] Association for the Development of Education in Africa (2006). (ADEA) A Toolkit for Mainstreaming Gender in Higher Education in Africa. Report of the Higher Education Working Group, Association of African Universities, Ghana.

[B9] Association for the Development of Education in Africa (ADEA) (2015). Tackling Gender Inequality in Higher Education Institutions in Africa: From Affirmative Action to Holistic Approaches. Policy brief, Forum of African Women Educationists (FAWE), Summit on Higher Education on Revitalizing Higher Education for Africa's future (Dakar, Senegal, March 10, 12, 2015).

[B10] Bassey S. A. Bubu N.G.C. (2019). Gender inequality in Africa: a re-examination of cultural values. Bucharest 11, 21–36. Available online at: https://www.ceeol.com/search/article-detail?id=884671 (accessed October 10, 2018).

[B11] Beaudry C. Prozesky H. St-Pierre C. Huet P. (2018) Factors that Affect Scientific Production in Africa: A Gender Analysis. Research Features. Available online at: http://researchfeatures.com/wp-content/uploads/2018/03/Catherine-Beaudry-Polytechnique-Montreal-Science-and-Tech.pdf (accessed: January 10, 2021).

[B12] Behning U Foden D Pascual A. S. (2001). “Introduction,” in Gender Mainstreaming in the European Employment Strategy, eds U. Behning, and A. S.Pascual (Brussels, European Trade Union Institute), 129–156.

[B13] Beveridge F. Nott S. Stephen K. (2000). Making Women Count - Integrating Gender into Law and Policy-making. Aldershot: Ashgate.

[B14] Chataway J. Dobson C. Daniels C. Byrne R. Hanlin R. Tigabu A. (2019). Science granting councils in Sub-Saharan Africa: trends and tensions. Sci. Public Policy 46, 620–631. 10.1093/scipol/scz007

[B15] Chataway J. Ochieng C. Byrne R. Daniels C. Dobson C. Hanlin R.. (2017). Case Studies of the Political Economy of Science Granting Councils in Sub-Saharan Africa. Full report, Canadian International Development Research Centre (IDRC), the UK Department for International Development (DfID) and South Africa's National Research Foundation (NRF) Science Granting Councils Initiative (SGCI). Available online at: https://idl-bnc-idrc.dspacedirect.org/handle/10625/56808 (accessed: October 10, 2021).

[B16] Council of Europe (1998). (CoE)Gender Mainstreaming: Conceptual Framework, Methodology and Presentation of Good Practices. Council of Europe: Strasbourg.

[B17] Daniels J. Nduati R. Kiarie J. Farquhar C. (2015). Supporting early career health investigators in Kenya: a qualitative study of HIV/AIDS research capacity building. Pan Afr. Med. J. 20:192. 10.11604/pamj.2015.20.192.5964 26113923 PMC4469515

[B18] Denney M. (2015). Gender and the Sustainable Development Goals: Moving beyond Women as a ‘Quick Fix' for Development. Center for Governance and Sustainability, 11. University of Massachusetts, Boston. Brief.

[B19] Department for International Development (DFID). (2019). Political Economy Analysis How to Note. Available online at: http://www.gsdrc.org/docs/open/po58.pdf (accessed October 10, 2018).

[B20] Elsevier B.V. (2017). Gender in the Global Research Landscape, Amsterdam: Elsevier B.V.

[B21] Escobar M. Forni L. Ghosh E. Davis M. (2017). Guidance Materials for Mainstreaming Gender Perspectives into Model-based Policy Analysis. Stockholm Environment Institute, California, U.S.A.

[B22] European Commission (EC) (2016). Strategic Engagement for Gender Equality 2016–2019. European Commission Directorate-General for Justice and Consumers, Brussels.

[B23] European Commission (EC) (2019). Report on Equality Between Women and Men in the EU. Luxembourg: Publications Office of the European Union. ISBN 978-92-76-00028-0

[B24] European Institute for Gender Equality (EIGE) (2016). Gender Mainstreaming Platform. Available online at: https://eige.europa.eu/publications/gender-mainstreaming-platform-leaflet (accessed: March 15, 2020).

[B25] Fitzgerald T. (2020) Mapping the terrain of leadership: gender and leadership in higher education. Ir. Educ. Stud. 39, 221–232. 10.1080/03323315.2020.1729222.

[B26] Fox M. F. (2020). Gender, science, and academic rank: key issues and approaches. Quant. Sci. Stud. 1, 1001–1006. 10.1162/qss_a_00057

[B27] Glanzel W. (2001). National characteristics in international scientific co-authorship relations. Scientometrics 51, 69–115. 10.1023/A:1010512628145

[B28] Glanzel W. (2002). Coauthorship patterns and trends in the sciences (1980–1998): a bibliometric study with implications for database indexing and search strategies. Libr. Trends 50, 461–473.

[B29] Glanzel W. Schubert A. (2004). “Analyzing Scientific Networks through Co-authorship,” in Handbook of Quantitative Science and Technology Research, ed H. Moed (The Netherlands: Kluwer), 257–276. 10.1007/1-4020-2755-9_12

[B30] Global Research Council (GRC) (2016a). Equality and Status of Women in Research. Survey Report of the Global Research Council 2016 Annual Meeting. Vitae, Oxford UK.

[B31] Global Research Council (GRC) (2016b). Case Studies of GRC Participants' Policies and Practices Relating to Gender Equality in Research. Survey Report of the Global Research Council 2016 Annual Meeting. Vitae, Oxford UK.

[B32] Global Research Council (GRC) (2017). Statement of Principles and Actions Promoting the Equality and Status of Women in Research. Supporting Women in Research: Policies, Programs, and Initiatives Undertaken by Public Research Funding Agencies. GRC Gender Working Group.

[B33] Goffman W. Harmon G. (1971). Mathematical approach to prediction of scientific discovery. Nature 229, 103–104. 10.1038/229103a0 16059101

[B34] Harding S. (2009). Postcolonial and feminist philosophies of science and technology: convergences and dissonances. J. Postcolonial Stud. 12, 401–421. 10.1080/13688790903350658

[B35] International Development Research Center (IDRC) (2018). Science Granting Councils Initiative of Sub-Saharan Africa (SGCI). Available online at: https://www.idrc.ca/en/initiative/science-granting-councils-initiative-sub-saharan-africa (accessed: October 10, 2018).

[B36] Jackson J. Neba A. Viney C. Mtwisha L. de-Graft Aikins A. Mitchel A.. (2022). Pathways to research leadership for early career researchers in Africa: a potential role for African and Global Funders. S. Afr. J. High. Educ. 36. (forthcoming).

[B37] Joshi A. Neely B. Emrich C. Griffiths D. George G. (2015). Gender research in AMJ: an overview of five decades of empirical research and calls to action. Acad. Manag. J. 58, 1450–1475. 10.5465/amj.2015.4011

[B38] Katz J. Hicks D. (1997). How much is a collaboration worth? A calibrated bibliometric model. Scientometrics 40, 541–554. 10.1007/BF02459299

[B39] Koseoglu M. (2016). Growth and structure of authorship and co-authorship network in the strategic management realm: evidence from the strategic management journal. BRQ Business Res Quart. 19, 153–170. 10.1016/j.brq.2016.02.001

[B40] Kraemer-Mbula E. Scerri M. (2016). Southern Africa. UNESCO Science Report: towards 2030. Paris, United Nations Educational, Scientific and Cultural Organization: 535–565.

[B41] Kraemer-Mbula E. Wamae W. (2010). Innovation and the Development Agenda, Organisation for Economic Co-operation and Development. Paris: IDRC Publishers, Ottawa.

[B42] Lee Y. A. Schottenfeld M. A. (2014). Collaborative knowledge creation in the higher education academic library. J. Learn. Spaces 3,1. Available online at: http://libjournal.uncg.edu/jls/article/view/714/550 (accessed October 10, 2018).

[B43] Liani M. L. Nyamongo I. K. Tolhurst R. (2020). Understanding intersecting gender inequities in academic scientific research career progression in sub-Saharan Africa. Int. J. Gend. Sci. Technol. 12, 262–288. 10.21203/rs.3.rs-479855/v1

[B44] L'Oréal Foundation (2018). Tectonic Movements - How Cultural Shifts Can Lift Up Women in Science. L'Oréal-UNESCO For Women in Science programme. Available online at: https://www.fondationloreal.com/media/1251/download (accessed: November 21, 2020)

[B45] Lundvall B.-A. Lema R. (2014). Growth and structural change in Africa: development strategies for the learning economy. Afr. J. Sci. Technol. Innov. Dev. 6, 455–466. 10.1080/20421338.2014.979660

[B46] Maphalala M. C. Mpofu N. (2017). Are we there yet? A literature study of the challenges of women academic in institutions of higher education. J. Gend. Behav. 15, 9216−9224. 10.520/EJC-b41b93666

[B47] Mauleón E. Bordons M. Oppenheim C. (2008). The effect of gender on research staff success in life sciences in the Spanish National Research Council. Res. Eval. 17, 213–225. 10.3152/095820208X331676

[B48] Mazey S. (2001). Gender Mainstreaming in the EU: Principles and Practice. London: Kogan Page.

[B49] Melin G. Persson O. (1996). Studying research collaboration using co-authorships. Scientometrics 36, 363–377. 10.1007/BF0212960030912999

[B50] Moed H. Glanzel W. Schmoch U. (2004). Handbook of Quantitative Science and Technology Research: The Use of Publication and Patent Statistics in Studies of SandT Systems. 1st Edn. New York, NY: Boston, London, Moscow: Kluwer. 10.1007/1-4020-2755-9

[B51] Moodly A. Toni N.M. (2017). Accessing higher education leadership: towards a framework for women's professional development. S. Afr. J. High. Educ. 31, 138–153. 10.20853/31-3-917

[B52] Mouton J. Gaillard J. van Lill M. (2015). “Functions of science granting councils in sub Sahara Africa,” in Knowledge Production and Contradictory Functions in African Higher Education, ed N. Cloete, P. Maasen, and T. Bailey (African Minds), 148–170.

[B53] Moya-Anegon F. Vargas-Quesada B. Chincilla-Rodriguez Z. Corera Alavarez E. Herrero-Solana Y. Munoz-Fernandez F. (2005). Domain analysis and information retrieval through the construction of heliocentric maps based on ISI-JCR category cocitation. Inf. Process. Manage. 41, 1520–1533. 10.1016/j.ipm.2005.03.017

[B54] Neimanis A. (2001). Gender Mainstreaming in Practice: A Handbook. United Nations Development Programme Regional Bureau for Europe and the CIS (UNDP RBEC).

[B55] New Partnership for Africa's Development (NEPAD) (2014). African Innovation Outlook 2. NEPAD Science, Technology and Innovation Hub, Pretoria, South Africa.

[B56] Okubo Y. (1997). Bibliometric Indicators and Analysis of Research Systems: Methods and Examples. 1st Edn. Paris, France: Organization for Economic Co-operation and Development.

[B57] Organization for Economic Cooperation Development (OECD) (2005). Guidelines for Collecting and Interpreting Innovation Data: Oslo Manual, 3rd Edn. Available online at: http://unstats.un.org/unsd/EconStatKB/Attachment336.aspx?AttachmentType=1 (accessed: Oct 24, 2021).

[B58] Parpart J. L. Connelly M. P. Barriteau V. E. (2000). Theoretical Perspectives on Gender and Development. International Development Research Centre, Ottawa, Canada

[B59] Payumo J. Sutton T. C. (2015). A bibliometric assessment of ASEAN's output, influence, and collaboration in plant biotechnology. Scientometrics 103, 1043–1059. 10.1007/s11192-015-1582-x

[B60] Payumo J.G. Monson J. Jamison A. Fenwick B. (2019). Metrics-based profiling of university research engagement with Africa: research management, gender, and internationalization perspective. Scientometrics 121, 675–698. 10.1007/s11192-019-03211-y

[B61] Prozesky H. Beaudry C. (2019) Mobility, gender career development in higher education: results of a multi-country survey of African Academic Scientists. Soc. Sci. 8:188. 10.3390/socsci8060188

[B62] Prozesky H. Mouton J. (2019) A gender perspective on career challenges experienced by African scientists. S Afr J Sci. 115, 1–5. 10.17159/SAJS.2019/5515

[B63] Raghupathi V. Raghupathi W. (2019). Exploring science-and-technology-led innovation: a cross-country study. J. Innov. Entrepreneurship 8:5. 10.1186/s13731-018-0097-0

[B64] Rigby J. (2009). Comparing the scientific quality achieved by funding instruments for single grant holders and for collaborative networks within a research system: some observations. Scientometrics 78, 145–164. 10.1007/s11192-007-1970-y

[B65] Rosas S. R. Kagan J. M. Schouten J. T. Slack P. A. Trochim W. M. (2011). Evaluating research and impact: a bibliometric analysis of research by the NIH/NIAD HIV/AIDS clinical trials networks. PLoS ONE 6:e17428. 10.1371/journal.pone.0017428 21394198 PMC3048860

[B66] Schiebiger L. (2010). Gender Science and Technology. Report of Expert Group Meeting. Paris: UNESCO. Available online at: https://www.un.org/womenwatch/daw/egm/gst_2010/Final-Report-EGM-ST.pdf

[B67] Schiebinger L. Schraudner M. (2011). Interdisciplinary Approaches to Achieving Gendered Innovations in Science, Medicine, and Engineering. Interdisciplinary Sci. Rev. 36, 154–67. 10.1179/030801811X13013181961518

[B68] Skupin A. (2009). Discrete and continuous conceptualizations of science: implications for knowledge domain visualization. J. Informetr. 3, 233–245. 10.1016/j.joi.2009.03.002

[B69] Small H. (2006). Tracking and predicting growth areas in science. Scientometrics 68, 595–610. 10.1007/s11192-006-0132-y 32214554 PMC7088887

[B70] Soderlund T. Madison G. (2015). Characteristics of Gender Studies Publication: A Bibliometric Analysis Based on a Swedish Population Database. Scientometrics. 105, 1347–1387. 10.1007/s11192-015-1702-7

[B71] Soete L. Schneegans S. Eröcal D. Angathevar B. Rasiah R. (2015). UNESCO Science Report Towards 2030 Focus on Sub-Saharan Africa, Paris, France: UNESCO Publishing.

[B72] Southern African Research and Innovation Management (SARIMA) (2017). Annual Report of the Science Granting Councils Initiative in Sub Saharan Africa (SGCI), Pretoria, South Africa.

[B73] Southern African Research and Innovation Management (SARIMA) (2018). Annual Report of the Science Granting Councils Initiative in Sub Saharan Africa (SGCI), Pretoria, South Africa.

[B74] Stvilia B. Hinnant C. C. Schindler K. Worrall A. Burnett G. Burnett K. Kazmer M. M. Marty P.F. (2011). Composition of scientific teams and publication productivity at a national science lab. J. Am. Soc. Informat. Sci. Tech. 62, 270–283. 10.1002/asi.21464

[B75] Tannenbaum C. Greaves L. Graham I. (2016). Why sex and gender matter in implementation research. BMC Med. Res. Methodol. 16:145. 10.1186/s12874-016-0247-7 27788671 PMC5084413

[B76] Tijssen R. Kraemer-Mbula E. (2017). Research excellence in Africa: policies, perceptions, and performance. Sci. Public Policy 45, 392–403. 10.1093/scipol/scx07434988271

[B77] UNESCO (2013). Mapping Research and Innovation in the Republic of Botswana. GO-SPIN Country Profiles in Science, Technology and Innovation Policy. Paris, UNESCO.

[B78] UNESCO (2021). UNESCO Science Report: The Race Against Time for Smarter Development. Paris, UN Educational, Scientific and Cultural Organization.

[B79] UNESCO. (2010). Gender Science and Technology. Report of Expert Group Meeting. Paris: UNESCO.Available online at: https://www.un.org/womenwatch/daw/egm/gst_2010/Final-Report-EGM-ST.pdf

[B80] United Nations (2011). Conference on Trade and Development. Applying a Gender Lens to Science, Technology and Innovation, Switzerland: United Nations.

[B81] United Nations (UN) (1997). Gender Mainstreaming. The Report of the Economic and Social Council for 1997. United Nations Headquarters, New York.

[B82] United Nations Women (2018a). Gender Mainstreaming Concepts and Definitions. Available online at: http://www.un.org/womenwatch/osagi/conceptsandefinitions.htm (accessed October 10, 2018).

[B83] United Nations Women (2018b). Turning Promises Into Action: Gender Equality in 2030 Agenda for Sustainable Development. Macedonia, OH: AGS Customs Graphics. Available online at: https://unstats.un.org/sdgs/files/meetings/iaeg-sdgs-meeting-07/11.2%20UN%20Women.pdf

[B84] Upham S. Small H. (2010). Emerging research fronts in science and technology: patterns of new knowledge development. Scientometrics 83, 15–38. 10.1007/s11192-009-0051-9 32214555 PMC7088980

[B85] Urama K. Muchie M. Twiringiyimana R. (2016). East and Central Africa. UNESCO Science Report: Towards 2030 (Paris: United Nations Educational, Scientific and Cultural Organization), 499–533.

[B86] van Eck N. J. Waltman L. (2018). Manual for VOSviewer Version 1.6.9.

[B87] Verloo M. (2001). Another Velvet Revolution? Gender Mainstreaming and the Politics of Implementation. IWM Working Paper No. 5/2001.

[B88] Wagner C. Leydesdorff L. (2005). Mapping the network of global science: comparing international co-authorships from 1990 to 2000. Int. J. Tech. Globalizat. 1, 185–208. 10.1504/IJTG.2005.007050

[B89] Walby S. (2005). Gender mainstreaming: Productive tensions in theory and practice. Social Politics 12, 321–343. 10.1093/sp/jxi018

[B90] Walby S. (2011). The Future of Feminism, Polity Press. Cambridge.

[B91] Woodward A. (2003). European gender mainstreaming: innovative policy or disappearing act'. Rev. Policy Res. 20, 65–88. 10.1111/1541-1338.d01-530052111

[B92] World Health Organisation (2002). (WHO) WHO Gender Policy: Integrating Gender Perspectives in the Work of WHO. World Health Organisation, Geneva, Switzerland.

[B93] Zosuls K. M. Miller C. F. Ruble D. N. Martin C. L. Fabes R. A. (2011). Gender development research in sex roles: historical trends and future directions. Sex Roles 64, 826–842. 10.1007/s11199-010-9902-3 21747580 PMC3131694

