# Supplementary figures and images for: Perspectives on Gender in Science, Technology, and Innovation: A Review of Sub-Saharan Africa's Science Granting Councils and Achieving the Sustainable Development Goals
Source: Front Res Metr Anal. 2022 Apr 11;7:814600. doi: 10.3389/frma.2022.814600 (PMC9035601; doi:10.3389/frma.2022.814600)

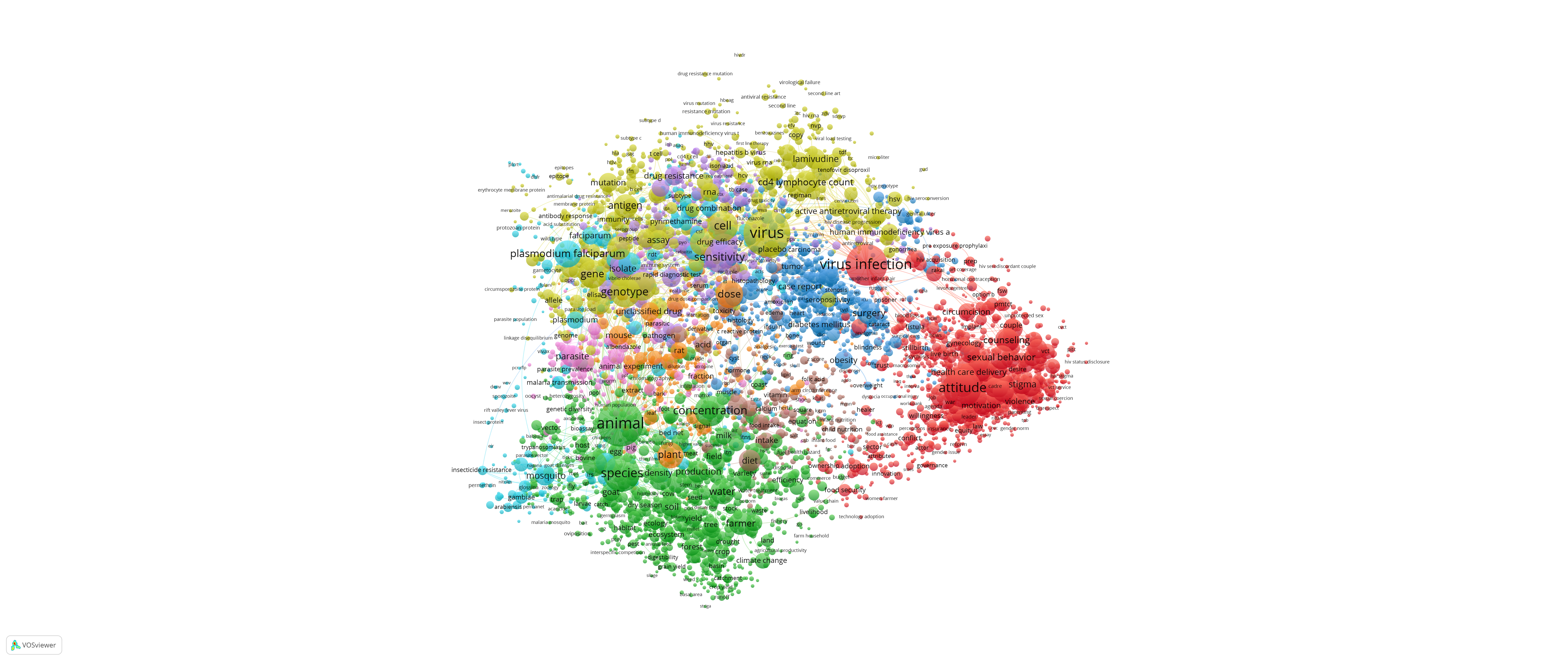

Supplement: Supplementary Figure 1 — Bibliometric analysis of the keywords in gender-related publications of SGCI-member countries. The size of nodes indicates the frequency of occurrence. The curves between the nodes represent their co-occurrence in the same publication. The shorter the distance between two nodes, the larger the number of co-occurrences of the two keywords. [file Image_1.PNG]
